# Binding to the conserved and stably folded guide RNA pseudoknot induces Cas12a conformational changes during ribonucleoprotein assembly

**DOI:** 10.1016/j.jbc.2023.104700

**Published:** 2023-04-12

**Authors:** Sruthi Sudhakar, Christopher L. Barkau, Ramadevi Chilamkurthy, Halle M. Barber, Adrian A. Pater, Sean D. Moran, Masad J. Damha, P.I. Pradeepkumar, Keith T. Gagnon

**Affiliations:** 1 Department of Chemistry, Indian Institute of Technology Bombay, Mumbai, India; 2 Department of Biochemistry and Molecular Biology, School of Medicine, Southern Illinois University, Carbondale, Illinois, USA; 3 Department of Chemistry, McGill University, Montreal, Quebec, Canada; 4 Department of Chemistry and Biochemistry, Southern Illinois University, Carbondale, Illinois, USA

**Keywords:** CRISPR-cas12a, evolution, induced fit, molecular dynamics, ribonucleoprotein assembly

## Abstract

Ribonucleoproteins (RNPs) comprise one or more RNA and protein molecules that interact to form a stable complex, which commonly involves conformational changes in the more flexible RNA components. Here, we propose that Cas12a RNP assembly with its cognate CRISPR RNA (crRNA) guide instead proceeds primarily through Cas12a conformational changes during binding to more stable, prefolded crRNA 5′ pseudoknot handles. Phylogenetic reconstructions and sequence and structure alignments revealed that the Cas12a proteins are divergent in sequence and structure while the crRNA 5′ repeat region, which folds into a pseudoknot and anchors binding to Cas12a, is highly conserved. Molecular dynamics simulations of three Cas12a proteins and their cognate guides revealed substantial flexibility for unbound apo-Cas12a. In contrast, crRNA 5′ pseudoknots were predicted to be stable and independently folded. Limited trypsin hydrolysis, differential scanning fluorimetry, thermal denaturation, and CD analyses supported conformational changes of Cas12a during RNP assembly and an independently folded crRNA 5′ pseudoknot. This RNP assembly mechanism may be rationalized by evolutionary pressure to conserve CRISPR loci repeat sequence, and therefore guide RNA structure, to maintain function across all phases of the CRISPR defense mechanism.

Conformational changes are the basis of protein and RNA folding from long biopolymers into biologically active molecules (1). The affinity, specificity, and stability of molecular interactions often scales with buried surface area (or number of molecular contacts) and the degree of surface complementarity (2). For RNA-guided enzymes, coevolution of protein and cognate RNA usually results in burial of significant portions of the RNA in the protein (3, 4, 5, 6, 7). Thus, conformational accommodations, primarily for the guide RNA, are an important mechanism for molding and shaping interactions. Archaeal box C/D ribonucleoprotein (RNP) complexes, for example, have been characterized to undergo substantial remodeling and induced fit during RNP assembly (4, 8, 9, 10, 11). Argonaute enzymes undergo minimal, although potentially important, conformational changes while the guide RNA is stretched and splayed through the middle of the protein (7, 12, 13).

CRISPR were first observed in bacteria in 1987 (14) and 1989 (15) as conserved repeat sequences with regularly spaced interrupting sequences. However, the significance of CRISPR loci remained unclear. In 2002, when these repeats were observed in other prokaryotes and CRISPR associated (Cas) protein-coding genes were found nearby, a unifying name of CRISPR was coined for these loci (16). It was later found that CRISPR repeats contained sequences that correspond to bacteriophage, suggesting an origin for the repeats (17, 18, 19). In 2006, it was suggested that these repeats act as an adaptive immune system to protect against viral invaders (20). This hypothesis was subsequently shown to be correct (21, 22). The study of CRISPR function and evolution quickly followed, leading to characterization of diverse RNA-guided CRISPR-Cas systems (23).

CRISPR systems act in three phases: adaptation, expression, and interference. During adaptation and expression, foreign DNA sequences are incorporated into CRISPR loci and then expressed as RNA for assembly into CRISPR-Cas RNP effector complexes (24, 25). Cas12a enzymes originate from type V CRISPR systems comprising the genes *cas12a*, *cas4*, *cas1*, *cas2*, and the repeat array (25). The conserved Cas1/Cas2 complex functions in spacer acquisition (26, 27). The adaptation complex comprises dimers of Cas2, which bridge pairs of Cas1 dimers, and binds potential protospacers to coordinate their incorporation into the CRISPR array. Cas1 is thought to have originated from transposon-like elements (termed casposons) which encode Cas1 homologs. Diverse Cas1 homologs exhibit low sequence conservation but retain a similar structure across the Cas1 phylogeny (28). Recently it has been shown that the Cas1/Cas2 complex in the type I-B system of *Haloarcula hispanica* binds the upstream-most repeat and leader at motifs of the CRISPR locus in a sequence and spacing-specific manner to facilitate new spacer integration (29). Cas12a has been shown to process its own CRISPR RNA (crRNA), cleaving upstream of the repeat sequence, which is a stem–loop structure referred to as the 5′ handle. These steps all rely on recognition of the CRISPR array repeat sequence, either at the DNA or RNA level, which serves as a common thread across adaptation, expression, and interference phases of the CRISPR defense mechanism. This offers a rationale for strong conservation of repeat sequences in CRISPR array loci. In contrast, other components, like the Cas12a protein, may be permitted to undergo greater divergence so long as they still recognize CRISPR arrays or crRNA and execute their respective phases.

During the interference phase, RNA-guided CRISPR-Cas effector enzymes find and pair to complementary nucleic acids, most often DNA, and catalyze phosphodiester bond cleavage to induce degradation (25, 30, 31). CRISPR effector enzymes are composed of one or two RNA molecules and an endonucleolytic Cas protein (16, 32, 33). During interference, CRISPR–Cas complexes will first identify candidate targets by transient Cas protein binding to a protospacer adjacent motif (PAM) DNA sequence (31). The crRNA guide sequence will then pair to the complementary protospacer sequence adjacent to the PAM (34). The class 2, type V CRISPR systems use a single effector enzyme, Cas12a, and a single 42 nucleotide long crRNA guide (35). Cas12a appears to have low off-target effects in gene editing applications (36) and possesses a T-rich PAM sequence requirement (35). Interestingly, CRISPR–Cas12a complexes exhibit a nonsequence-specific, multiturnover single-stranded DNase activity, called *trans* activity, after sequence-specific cleavage of target DNA has occurred (37, 38). For this reason, Cas12a has been used to develop diagnostic assays, such as for COVID-19 detection (38). In addition, CRISPR–Cas12a is being adapted for human therapeutics (39, 40, 41).

Conformational changes that occur during CRISPR-Cas RNP assembly and their effects on enzyme activity have been investigated previously. The Doudna laboratory observed global structural changes during RNA binding to Cas9 (42, 43) that coincide with catalytic competence and have been supported by cryo-EM studies (44). Other studies have investigated RNP assembly and conformational dynamics for Cas9 (42, 45, 46). Several Cas enzyme crystal structures have been solved (5, 6, 47, 48, 49, 50, 51). Recent cryo-EM studies of Cas9 during precatalytic or target binding steps have revealed some new conformational states, yet these involve substrate DNA, which is difficult to uncouple from guide RNA-induced changes (44, 52). These structures provide important insight into CRISPR-Cas9 structure-function, however the full picture of RNP assembly dynamics may still be incomplete. Cas12a dynamics during RNP assembly with its cognate guide have been less explored, owing in part to the historical lack of available high-resolution structures of Cas12a in the absence of a guide RNA (53, 54). Cas12a dynamics studies have identified large-scale “open” and “closed” conformational states upon crRNA binding that appear to depend on the originating species of the CRISPR-Cas12a system (51, 53, 55). These studies focused on one Cas12a and large-scale global structural changes with target DNA substrate binding (55, 56).

In this study, we sought to investigate Cas12a RNP assembly through protein and RNA sequence and structural alignments, phylogenetics, molecular dynamics (MD) simulations, and spectroscopic and biochemical experiments. We find that Cas12a sequence diverged during evolution, although protein structure was relatively well-conserved in the RNP state. In contrast, the sequence and structure of the crRNA 5′ handle remained highly conserved. MD simulations of *Acidaminococcus* sp. strain BV3L6 (As) Cas12a, as well as Cas12a from *Lachnospiraceae bacterium* ND2006 (Lb) and *Francisella tularensis* subsp. *novicida* (Fn), identified substantial protein structural dynamics that were reduced upon RNA binding, especially in the Wedge domain involved in RNA and DNA binding. In contrast, the crRNA 5′ handle structure showed minimal variation between free and bound states. In-solution limited proteolysis followed by MS identified AsCas12a lysine residues with reduced accessibility after RNP assembly. Differential scanning fluorimetry showed improved thermal stability and folding cooperativity of AsCas12a as an RNP. For the crRNA 5′ handle, thermal denaturation and CD analyses suggested stable independent folding in the free state. Interdomain angle calculations from MD simulations predict little or no sampling of the RNP conformational state in apo-Cas12a, suggesting an induced fit over conformational selection model. Together, these results indicate that Cas12a protein undergoes substantial conformational rearrangements induced by binding to the largely prefolded crRNA 5′ pseudoknot structure. We propose that this assembly mechanism may be common for CRISPR-Cas systems due to the intrinsic need to conserve CRISPR loci repeat sequences and therefore crRNA structures.

## Results

**Figure 1:**
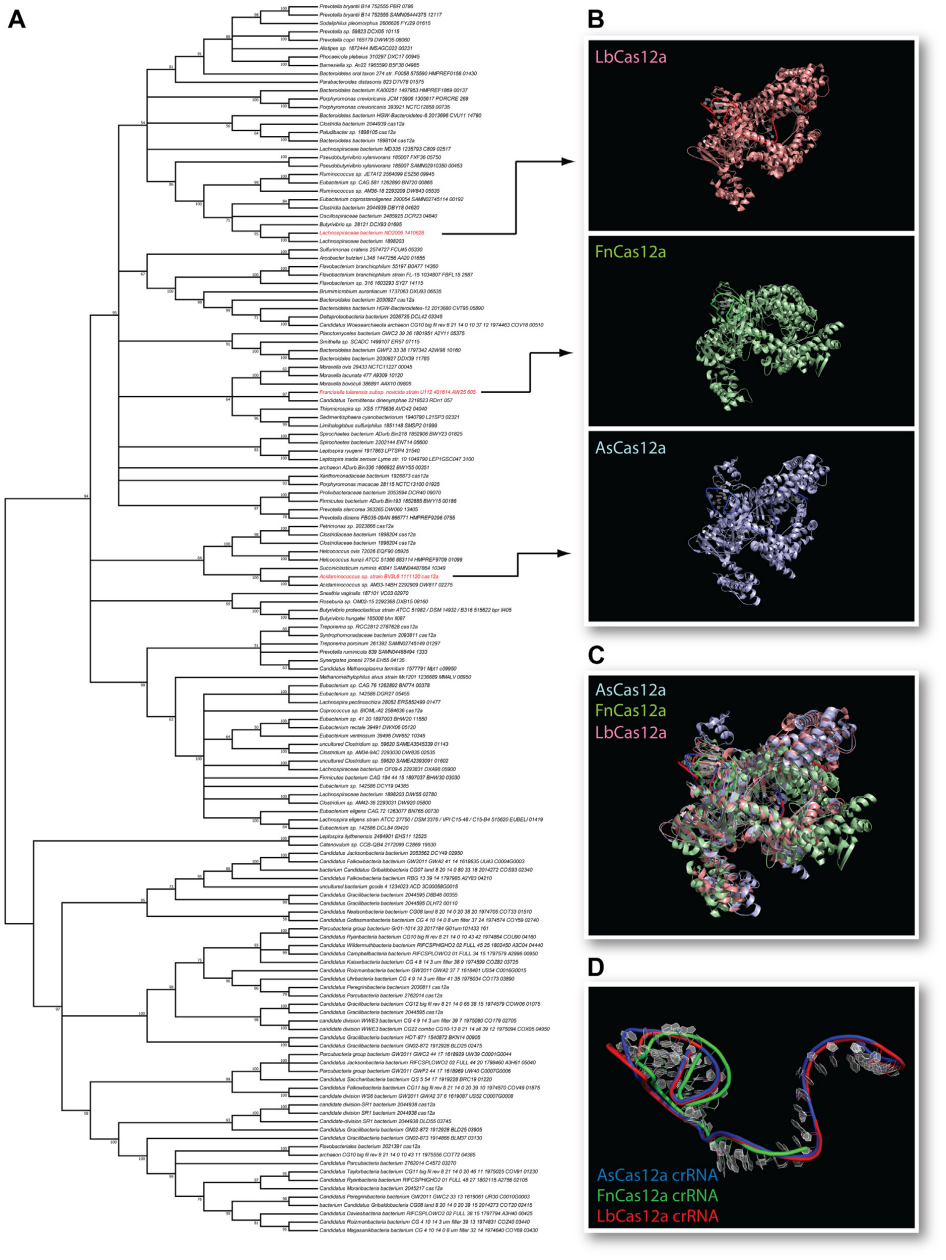
**Sequence and structural relationships among Cas12a homologs.***A*, consensus maximum likelihood tree of available Cas12a protein sequences. Structurally characterized homologs are labeled in *red*. *B*, individual crystal structures of Lb, Fn, and AsCas12a corresponding to their location on the tree shown in panel *A*. Structural alignment of As (PDB ID: 5B43), Fn (PDB ID: 5NG6), and Lb (PDB ID: 5XUS) Cas12a proteins (*C*) and crRNAs (*D*). As, *Acidaminococcus* sp; Fn, *Francisella tularensis* subsp. *Novicida*; Lb, *Lachnospiraceae bacterium*.

To understand RNP assembly across CRISPR-Cas12a systems, we began by performing a maximum likelihood phylogenetic analysis on an alignment of 147 publicly available Cas12a protein sequences. In our resulting consensus tree (Fig. 1*A*), most positions in the alignment were poorly conserved (∼39% on average at positions homologous to the AsCas12a homolog), leading to low fine-grain phylogenetic resolution. Three of the Cas12a proteins in the alignment have been structurally characterized previously, AsCas12a (6), FnCas12a (57), and LbCas12a (51). The structures of these homologs are shown in Figure 1*B* with their placement on the phylogenetic tree indicated. The resulting topology strongly suggests that among these three Cas12a homologs, FnCas12a and LbCas12a are more similar to each other than to AsCas12a. Overall, their sequence conservation is moderate based on alignment and BLAST (Fig. S1), with sequence identity/similarity being 40%/59% for LbCas12a to FnCas12a, 34%/50% for LbCas12a to AsCas12a, and 35%/51% for AsCas12a to FnCas12a (35, 51). Their structure, by contrast, is well-conserved, with pairwise RMSD values calculated at 2.56 Å for LbCas12a to FnCas12a, 2.20 Å for LbCas12a to AsCas12a, and 2.78 Å for AsCas12a to FnCas12a when assembled as RNP complexes (FnCas12a) or ternary complexes with guide RNA and target DNA (LbCas12a and AsCas12a) (Fig. 1*C*). These results suggest that the structure-function of Cas12a RNPs is conserved despite substantial protein sequence divergence.

**Figure 2:**
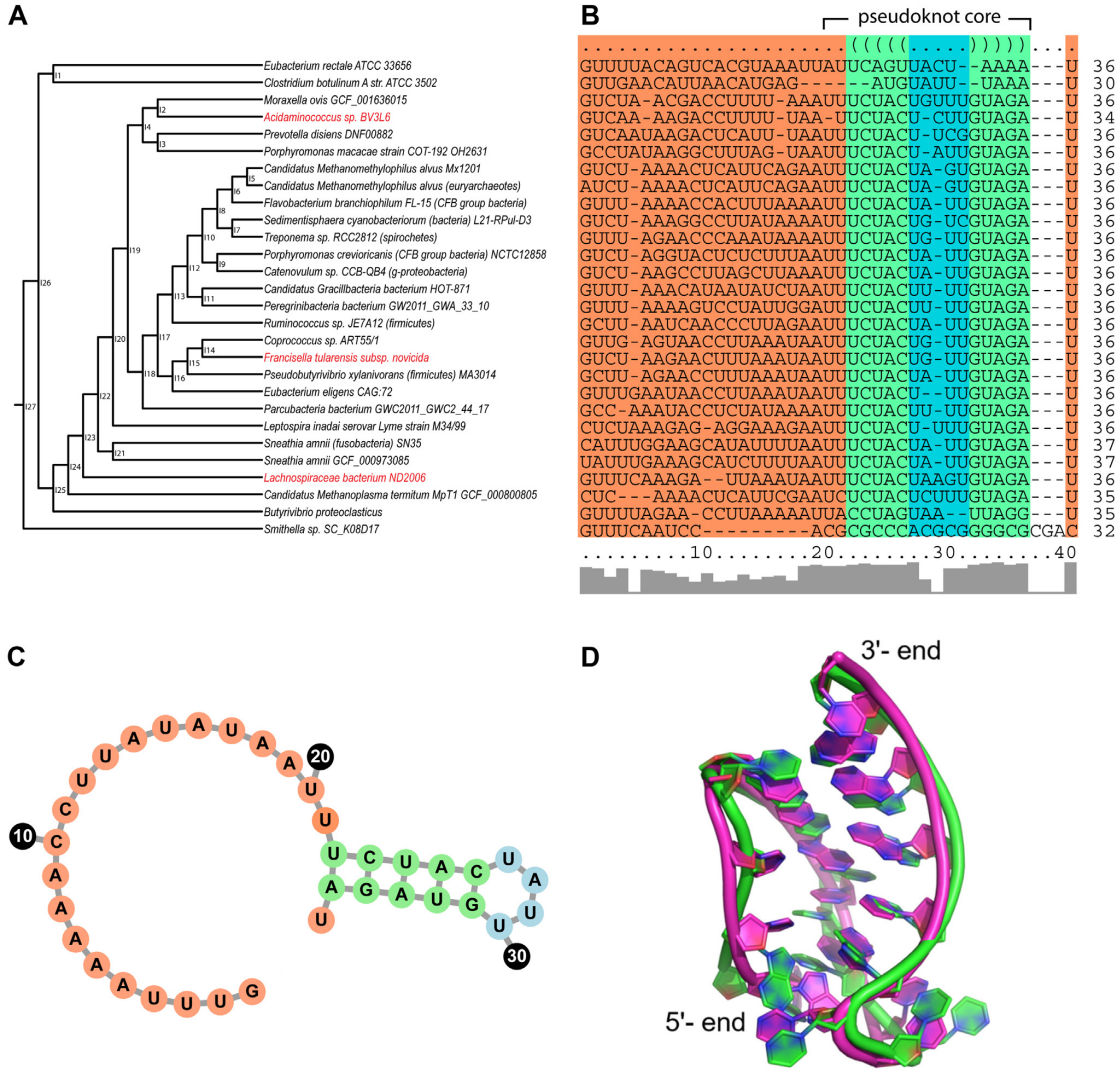
**Conservation of Cas12a-associated repeat sequence and folding.***A*, nearest neighbor joining tree and (*B*) alignment of 28 type V CRISPR repeats. The LocARNA-predicted secondary structure (*right*) is indicated at the *top* of the sequence alignment. The 5′ leader sequence is highlighted in *orange* and the stem and loop of the predicted stem–loop structure is highlighted in *green* and *blue*, respectively. *C*, repeat consensus sequence shown with the secondary structure predicted for the alignment in (*A*) with the same color coding. *D*, superimposed image of the representative structures of the major cluster in the free pseudoknot (*magenta*) and AsCas12a-bound pseudoknot (*green*) in the AsCas12a RNP complex from 1 μs molecular dynamics simulation trajectories. RNP, ribonucleoprotein.

RMSD values calculated between the crRNAs of each homolog revealed striking structural similarity (Fig. 1*D*), with the distances of AsCas12a to LbCas12a and FnCas12a crRNAs being 0.12 Å and 0.17 Å, respectively. The distance between LbCas12a and FnCas12a crRNAs was 0.21 Å. We also aligned repeat sequences from 28 type V CRISPR systems. In contrast to the Cas12a proteins, these sequences were highly conserved in the pseudoknot folding repeat region (35) (Fig. 2, *A* and *B*). The relative distance relationship observed among LbCas12a, FnCas12a, and AsCas12a proteins (Fig. 1*A*) was recapitulated in the topology of the nearest-neighbor tree of their cognate repeat crRNA sequences constructed by LocARNA (58). Secondary structure analysis of this alignment using LocARNA, which does not predict pseudoknots, revealed a conserved hairpin structure (35) (Fig. 2*B*) consistent with the core of the 5′ pseudoknot observed in solved Cas12a structures. The predicted structure is shown with the consensus sequence of the aligned repeats (Fig. 2*C*). These results suggest that the sequence and structure of the crRNA 5′ handle has remained remarkably stable.

To gain insights into the structural dynamics of Cas12a-crRNA RNP assembly, MD studies were conducted. Three different systems were initially prepared–AsCas12a protein alone (apo-AsCas12a), the AsCas12a RNA pseudoknot alone (free pseudoknot), and AsCas12a bound to its cognate crRNA (AsCas12a RNP)–and then subjected to 1 μs of an unrestrained production run in the graphics processor unit accelerated version of Particle Mesh Ewald Molecular Dynamics (59, 60, 61) in AMBER 18 (https://ambermd.org/) (62).

The analysis of trajectories was carried out in two stages. The pseudoknot structural stability and flexibility were explored in the first stage and the protein structural stability and flexibility were examined in the second stage. The RMSD of the pseudoknot backbone atoms were calculated using the first simulation frame as reference (Fig. S2*A*). The RMSD plot indicates minimal deviations between the free pseudoknot alone and the pseudoknot in the RNP. This suggests that the simulations are equilibrated and the pseudoknot is stable throughout the 1 μs production run. The RMSD value is slightly higher in the case of free pseudoknot, mainly due to the fraying of the terminal nucleotides and the absence of interactions with the protein. This is further demonstrated by the RMS fluctuations (RMSF) values (Fig. S2*B*). The RMSF plot shows that the residues exhibit similar fluctuations, with nearly identical values in the core hairpin of the structure, indicating that the pseudoknot behaves similarly in the free and bound forms.

The end-to-end distances in the pseudoknot can be used to report structural rigidity and stability during the simulation. The terminal nucleotides were not used for the measurement to avoid possible fluctuations due to the terminal fraying of base pairs. When the end-to-end distances were calculated, the values showed negligible deviations in the free and bound forms (Fig. S2*C*). Thus, the rigidity of the pseudoknot is maintained in both free and bound forms. The 1 μs simulation trajectories were clustered into ten ensembles. The representative structures of the major cluster of the free and bound pseudoknot were then superimposed to visualize structural fluctuations (Fig. 2*D*). The structures superimposed with an RMSD value less than 2.5 Å, confirming that the pseudoknot does not undergo any significant structural changes when bound to the Cas12a protein.

**Figure 3:**
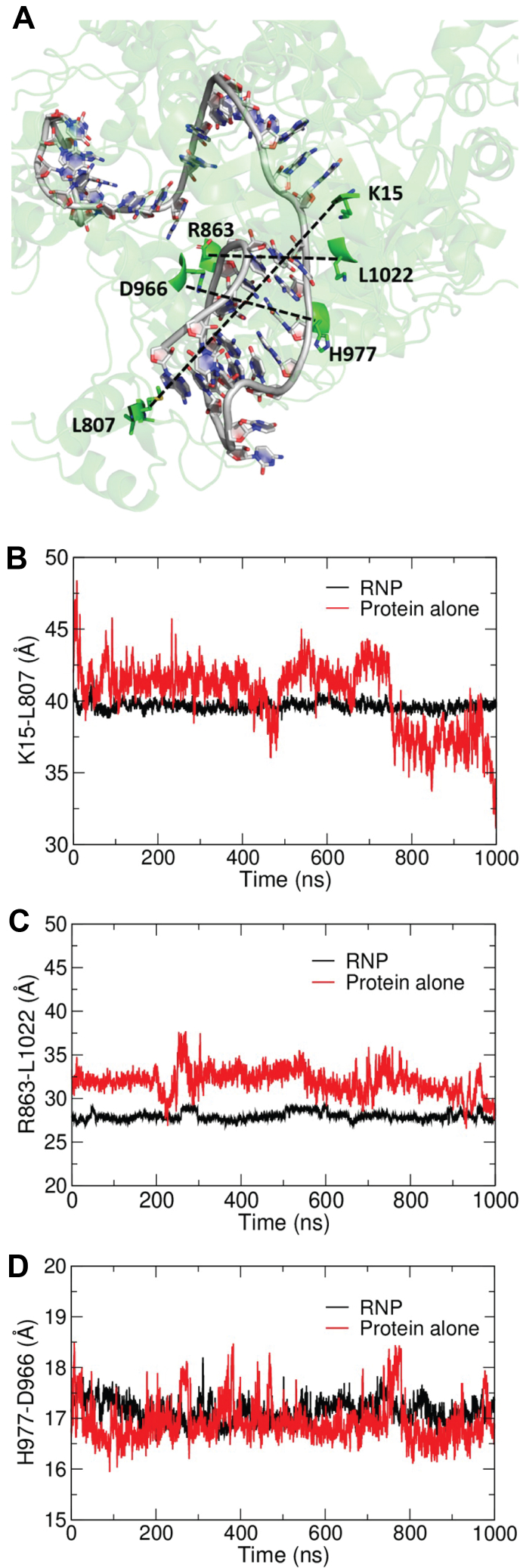
**Molecular dynamics simulations indicate that AsCas12a is structurally less dynamic and less solvent accessible when bound to its cognate crRNA.***A*, representative image illustrating the amino acids considered for distance measurements during molecular dynamic simulations. The distance between the Cα atoms of (*B*) K15 and L807, (*C*) R863 and L1022, and (*D*) H977 and D966 from 1 μs trajectories. The running averages of the distances are represented in the plot.

In the second stage of analysis, the RMSD of the backbone atoms of AsCas12a protein were measured with respect to the initial frame of the simulation (Fig. S3*A*). The equilibration of the simulations was observed from the RMSD plots, which showed marginal differences in the protein structure alone without the crRNA and in the RNP complex. The RMSF plots indicate that several regions, which include residues ranging from 150 to 300 and 750 to 85 show significant deviations between the protein alone *versus* in the RNP complex (Fig. S3*B*). These regions include parts of the PI, wedge, and REC domains. A previous study reported increased occupancy of a “closed” high fluorescence energy transfer (FRET) state for FnCas12a upon crRNA binding, as indicated by REC and NUC domain distance measurements *via* single molecule FRET (55). The RMSF values for the protein in the RNP complex were less than the protein alone without the pseudoknot, indicating that the protein becomes more ordered in the presence of the crRNA. The distance between the Cα atoms of amino acids in close vicinity of the pseudoknot were calculated to identify any rearrangements in the protein that might help accommodate the crRNA pseudoknot. We selected K15-L807, R863-L1022, and H977-D966 distances as representative since they are all in the wedge and RuvC domains and make close contact with either the loop or the terminals of the pseudoknot (Fig. 3*A*). The distances varied between the systems, and the protein in RNP showed smaller distance fluctuations than the protein alone, especially for K15-L807 and R863-L1022, which suggests conformational sampling by AsCas12a protein to accommodate the crRNA pseudoknot during RNP assembly (Fig. 3, *B*–*D*).

**Figure 4:**
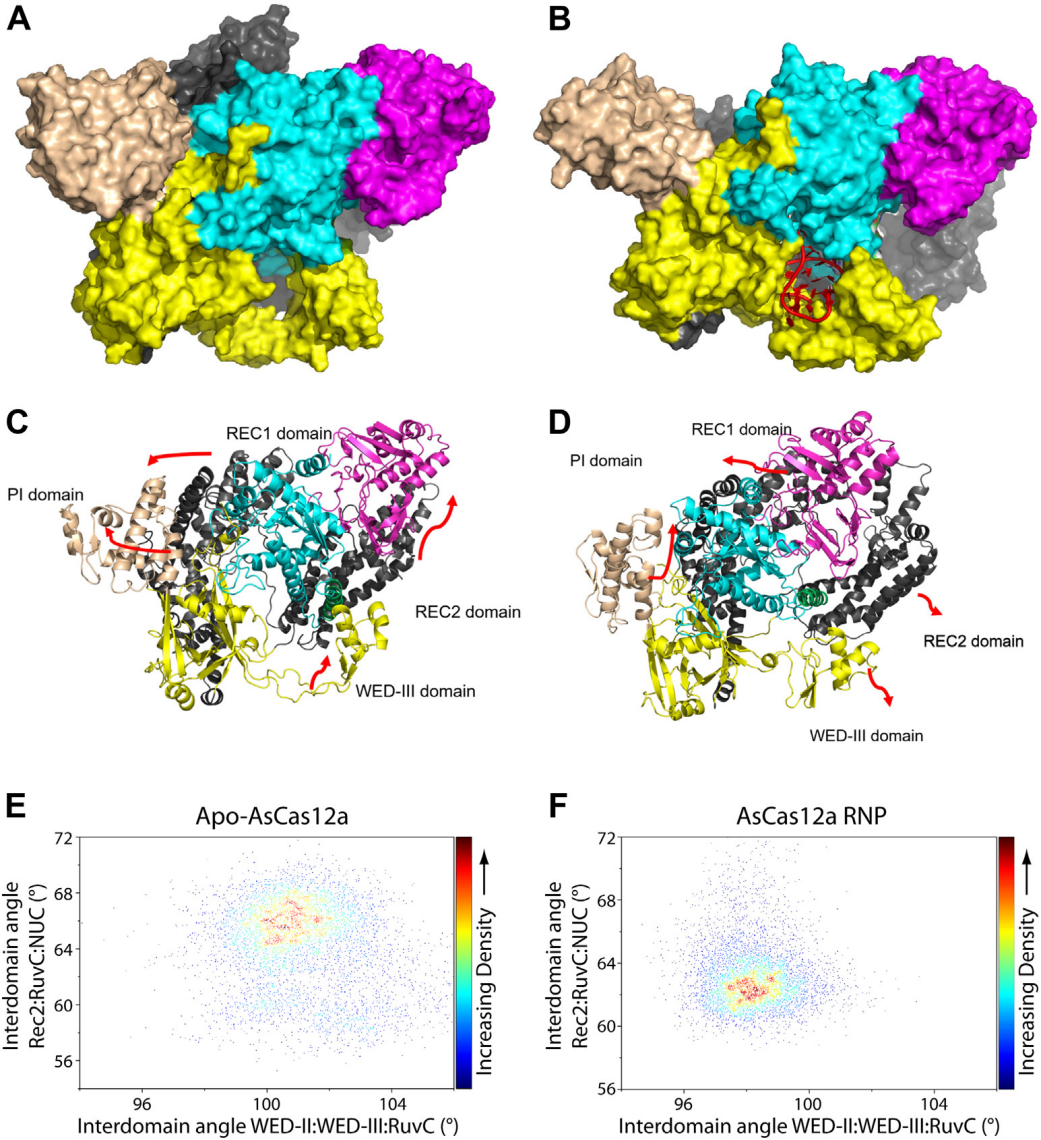
**Molecular dynamics simulations predict distinct dynamics and domain motion for AsCas12a protein *versus* AsCas12a RNP.** Representative snapshots of the major clusters of (*A*) AsCas12a protein system and (*B*) AsCas12a RNP complex from the 1 μs trajectories. The protein is shown in surface representation. The PI domain is represented in *beige color*, the wedge domain in *yellow*, RuvC domain in *cyan*, REC domain in *gray*, and the Nuc domain in *magenta*. The pseudoknot is represented as cartoon in *red*. Representative images of the principal motion of protein in (*C*) AsCas12a protein alone and (*D*) AsCas12a RNP complex. Domain colors are the same as in panels *A* and *B* except the bridge helix (BH) is shown in *green*. The *arrows* are marked according to the pseudo trajectory and do not indicate the amplitude of motion. The 3D perspective of the domain movements might not be clearly visible in the given representation. Density plots of the interdomain angles in (*E*) apo-AsCas12a protein alone or (*F*) AsCas12a RNP. The density plot represents the sampling of the angle and the maximum population is represented with the highest density. RNP, ribonucleoprotein.

The 2D-RMSD values were calculated for each protein domain to detect the regions of high protein mobility. The values clearly showed that the REC domain and WED-III domain showed significant differences between the two systems. The 2D-RMSD values of the wedge domain in the protein without pseudoknot RNA displayed values as high as 10 Å, while the values remained <5 Å in the case of the RNP (Fig. S4, *A* and *B*). This confirms our previous observation from the RMSF values that the protein becomes more stabilized in the presence of the crRNA. The representative structures of the major clusters (Fig. 4, *A* and *B*) were visually inspected to validate the variations seen in the 2D-RMSD plots. The WED-III domain and REC domain rearranged to incorporate the RNA into the RNP complex. The pseudoknot rearranges the WED-III domain while the single-stranded guide region rearranges the REC domain in the RNP complex for enhanced binding.

To identify the major mode of domain motion (maximum variance), principal component (PC) analysis was carried out (Fig. 4, *C* and *D*). The translational motion was initially removed by fitting the trajectory to an average structure. The projection diagrams and pseudo trajectories from PC analysis were analyzed to obtain a pattern of the major movements resulting in the conformational preferences discussed in the cluster analysis. In the protein only system, the REC2 and the WED-III directions allow them to occupy some of the space where the RNA is usually bound. Compared to the RNP complex, the REC2 and WED-III domains show movement directed toward creating space to accommodate the crRNA pseudoknot and guide spacer sequence. The PI and REC1 domains in the RNP complex exhibit a circular motion with one part slightly moving away from the cleft, while the smaller helix moves toward the cleft, which would subsequently bind the DNA target for catalysis (56). This motion is not exactly the same in the absence of crRNA. The amplitude of motion shows minimal variations in domains other than the PI in the first PC. The histogram of the first and second PC modes of the RNP and protein alone (Fig. S5*A*) shows no convergence suggesting the variation in conformational preferences. Though the deviation is not high, the regions spanned by the PC mode 1 and mode 2 of the apo protein and RNP complex are different (Fig. S5*B*).

Solvent accessible surface area (SASA) of the protein was calculated from the trajectories of protein alone and the RNP complex simulations (Fig. S6). The ΔSASA was found to be ∼4000 Å^2^, with the RNP complex having a less value, suggesting the protein becomes more ordered and compact upon RNP assembly. The rearrangements in the protein to accommodate RNA were explored further by comparing the surface electrostatic potential images of the protein (Fig. S7). The positively charged regions are rearranged in the RNP complex, likely to ensure better interaction with the negatively charged backbone of the RNA.

To rule out potential bias by using a manually generated model of apo-AsCas12a from a DNA-bound ternary structure, MD simulations were also carried out on apo form deposited in the AlphaFold database. Though the RMSF values were slightly lower than the manually generated structure, the overall trend in the structural properties (RMSD, RMSF, distances, and SASA) remained the same in both models (Figs. S8–S11).

MD simulations on FnCas12a (PDB ID: 5NG6) and LbCas12a (PDB ID: 5ID6) RNP, apoprotein, and pseudoknot were also carried out to determine whether our observations for AsCas12a might apply more broadly to other CRISPR–Cas12a complexes. For both FnCas12a and LbCas12a, the RNA pseudoknot was stable throughout the simulations with similar values of RMSF (Fig. S12) and end-to-end distances (Fig. S13) in both the free and bound forms. Due to terminal and loop nucleotide fraying, the RMSD is slightly higher for the free forms in both cases (Fig. S14). The major clusters of the free and bound form of pseudoknot superimposed with an RMSD value <2.7 Å and <2.6 Å in FnCas12a (Fig. S15*A*) and LbCas12a (Fig. S15*B*) complexes, respectively. Similar to AsCas12a, the pseudoknot structure also remained stable in these complexes.

The protein alone was more dynamic than the RNP complex for both FnCas12a and LbCas12a, with a higher value for the latter. In the FnCas12a system, the RMSF plots revealed more deviations in the wedge, PI, and RuvC domains, similar to what was observed in AsCas12a (Fig. S16*A*). The domain designations are different in LbCas12a but greater deviations in the RMSF values were found in the oligonucleotide binding domain (OBD) and RuvC domain (Fig. S16*B*). These results show that the domains responsible for interacting with RNA exhibit variation in dynamics during RNP assembly. The distance analysis between the Cα atoms of the amino acids in the immediate vicinity of the RNA pseudoknot revealed that the Cas12a protein had undergone some rearrangement to accommodate the pseudoknot in both FnCas12a and LbCas12a complexes (Fig. S17). While the values are constant in the RNP complexes, variations in the apo form were observed that indicate a compaction of the protein upon RNA binding.

The superimposition of major clusters of Fn and Lb Cas12a in the apo and RNP complexes revealed changes in the domain indicated by the RMSF values. While the WED-III domain loop showed maximum variation in FnCas12a (Fig. S18*A*), the OBD, UK, and RuvC domains showed high fluctuation in LbCas12a (Fig. S18*B*). Irrespective of the Cas12a type, the rearrangement is nearly identical around the RNA pseudoknot. Further, the electrostatic surface potential images also confirmed the rearrangement of positively charged residues to better accommodate the negatively charged backbone of the RNA pseudoknot (Fig. S19). In FnCas12a and LbCas12a, the RNP assembly results in about a 5% reduction in the overall SASA (Fig. S20). Overall, MD studies of FnCas12a and LbCas12a led to results quite similar to those of AsCas12a.

To assess whether conformational changes associated with the Cas12a RNP assembly are a result of induced fit or conformational selection, two related but distinct binding modes, interdomain angles from AsCas12a simulations were sampled (63). The RNA pseudoknot and single-stranded portion of the guide RNA are near the Wedge, REC, RuvC, and NUC domains. Therefore, the orientation of these domains might differ in the apo form and RNP complex. The interdomain angles between WED-II: WED-III: RuvC (Fig. S21*A*) and REC2: RuvC: NUC (Fig. S21*B*) were calculated (RuvC-I and RuvC-II were considered together). The density plots with these angles as coordinates revealed that the sampling differs in the apo and RNP forms (Fig. 4, *E* and *F*). The apo form exhibits an open structure with a large interdomain angle, while the RNP exhibits a closed structure with a slight interdomain angle. The angle associated with the closed form is only seen in the RNP and not present in the sampling of the apo-protein. The cluster distribution of the apo and RNP also confirmed the same. The major clusters of the apo and RNP forms show high RMSD upon superimposing with each other and no representative structures from the clustering are similar in both of these forms (Fig. 4, *A* and *B*). Thus, the closed form is associated only with the RNP and the apo form does not exhibit the flexibility to adopt open/closed conformations. These results suggest that Cas12a RNP assembly proceeds through induced fit rather than conformational selection since the necessary RNP conformation is essentially never sampled in the apo form (63).

**Figure 5:**
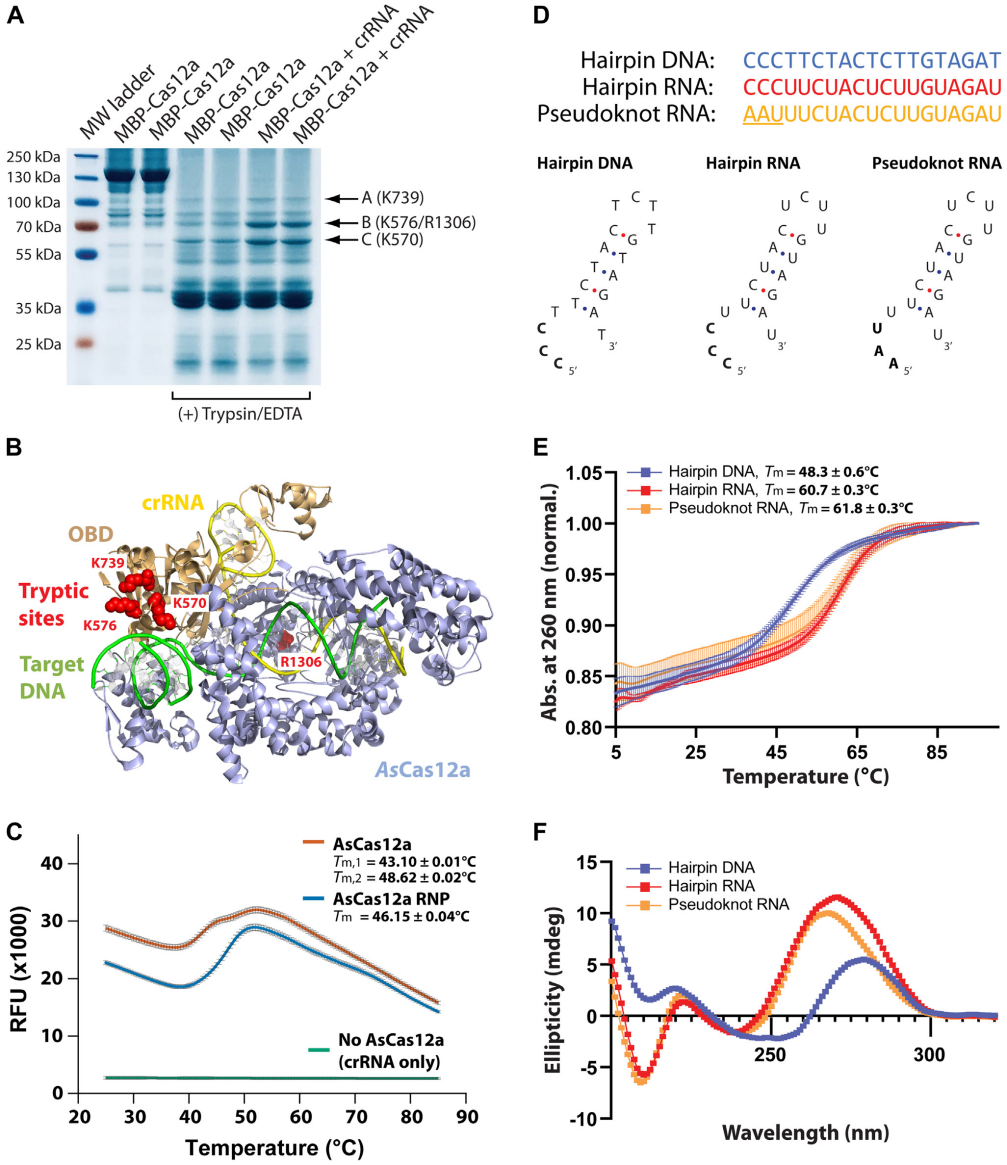
**Cas12a undergoes conformational changes upon RNP assembly, whereas the crRNA pseudoknot is independently folded.***A*, limited trypsin proteolysis of AsCas12a with and without its cognate crRNA bound. Digestion products corresponding to differential trypsin accessibility are indicated to the right. *B*, amino acids corresponding to differentially accessible sites from (*A*), determined by MS, are highlighted in *red spheres* on the structure of AsCas12a. *C*, differential scanning fluorimetry of AsCas12a alone or bound to crRNA. *D*, sequence and predicted 2D structure of RNA and DNA used for thermal denaturation and CD analyses. *E*, UV-monitored thermal denaturation analysis and (*F*) CD spectra of the AsCas12a 5′ handle pseudoknot unbound to AsCas12a. DNA and RNA hairpins are comparative controls. Error is shown as SEM of three experimental replicates. RNP, ribonucleoprotein.

To experimentally investigate the structural flexibility of AsCas12a, we subjected the free protein or assembled RNP to limited trypsin proteolysis. The presence of crRNA appeared to reduce cleavage efficiencies at three sites (Fig. 5*A*). Mass spectrometric analysis of these bands revealed that they corresponded to protection from trypsin proteolysis at K739, K570, and K576 (Fig. S22). Of these, K570 and K576 are found in the OBD-II, which is involved in crRNA and target DNA binding (51) (Fig. 5*B*). To observe global effects on AsCas12a conformation and stability during RNP assembly, we performed differential scanning fluorimetry (DSF) on AsCas12a with and without crRNA. This method uses a dye specific for protein rather than UV absorbance at 280 nm, eliminating potentially confounding UV signal overlap from the RNA. AsCas12a alone exhibited a biphasic melt profile, suggesting at least two structures that largely unfolded independently (64). Their melting temperature (*T*_m_) values were calculated at 43.10 °C ± 0.01 deg. C and 48.62 °C ± 0.02 deg. C. When crRNA was present, AsCas12a unfolded cooperatively as an apparent single structure with a *T*_m_ of 46.15 °C ± 0.04 deg. C (Fig. 5*C*). To assess the unfolding model that best fits the data, we performed fits to a two-state folded–unfolded model and to a three-state sequential folded-intermediate-unfolded model (Fig. S23). While this model fitting is not sufficient to provide a detailed understanding of the protein folding landscape, unfolding *via* a structural intermediate in the three-state model fit the data best. This suggests that significant structural or stability changes to Cas12a occurred upon RNP assembly.

To determine the stability and folding of the crRNA pseudoknot in solution, we performed UV-monitored thermal denaturation and CD analyses. The RNA pseudoknot sequence is composed of the 19 nucleotides of the AsCas12a crRNA 5′ end. Control DNA and RNA hairpin sequences were created by replacing the three 5′ terminal nucleotides with cytidines to prevent pseudoknot formation (Fig. 5*D*). Thermal denaturation revealed that the pseudoknot unfolded similarly to the control, model hairpin RNA sequence, with similar *T*_m_ values of 61.8 °C ± 0.3 deg. C and 60.7 °C ± 0.3 deg. C, respectively (Fig. 5*E*). The slight increase in *T*_m_ for the pseudoknot may be attributable to additional base-pairing interactions in the structure (65). As expected, the DNA hairpin appeared to possess a less stable structure (65), with a *T*_m_ of 48.3 °C ± 0.6 deg. C. When we performed CD analyses, we observed nearly identical spectra for the pseudoknot RNA and hairpin RNA, characteristic of A-form structure (66), with a small shift in the 250 to 300 nm range (Fig. 5*F*). The DNA hairpin generated a distinct spectra indicative of B-form structure (66). These results point to a well-formed crRNA pseudoknot in solution independent of protein binding.

## Discussion

Our results indicate that Cas12a proteins undergo conformational rearrangements to accommodate crRNA binding during RNP assembly. In contrast, the crRNA 5′ pseudoknot, the primary “handle” that Cas12a binds, appears to undergo very little structural alterations. The highly conserved nature of the crRNA repeat sequence, corresponding to the 5′ pseudoknot handle, and its interconnected role across adaptation, expression, and interference phases of CRISPR mechanism, implies that alterations to sequence or structure would likely be deleterious to the system. Cas12a proteins, on the other hand, appear to evolve much more rapidly, sustaining far more sequence changes, and to a lesser extent structural alterations, through evolution. In-solution studies demonstrated conformational changes of AsCas12a upon crRNA binding while the crRNA pseudoknot handle itself appeared quite stable alone, in agreement with MD simulations.

RNA-guided enzymes are a unique class of enzymes that share an intimate relationship with their cognate RNA partners. The protein component is typically inactive when not associated with its RNA guide (67). The guide RNA specifies the substrate through Watson–Crick hybridization and correctly positions it into the protein's active site (7, 68). The guide sequence can vary, allowing a single type of protein to catalyze the same reaction on countless targets of varying sequence. Since RNA-guided enzymes can be programmed to target different sequences, understanding the rules of RNP assembly is important for understanding how these systems evolved and how to better leverage them for biotechnology and biomedical applications.

For engineering Cas12a enzymes, it is important to not introduce modifications that can significantly perturb the folding or stability of the pseudoknot structure. A recent study investigated a diverse class of chemical modifications in the pseudoknot and indeed concluded that smaller modifications known to maintain RNA structure, such as 2′-fluororibose, were the most compatible (39). Likewise, targeted mutagenesis of Cas12a protein should avoid altering the dynamic flexibility or folding of the enzyme, such as increasing rigidity, in regions that must mediate guide RNA contact or else risk weaker binding affinity and poor RNP assembly or stability. Interestingly, unbiased Cas12a mutagenesis screens aimed at improved activity found that favorable mutations lie outside of the wedge domain, which was the most dynamic during our crRNA binding simulations (41, 69).

These findings for Cas12a may also extend to other CRISPR-Cas systems. The need to conserve CRISPR array repeat sequences across all phases of the CRISPR mechanism is a common feature for CRISPR-Cas systems. For Cas9 systems, the structure of Cas9 from *Streptococcus pyogenes* has a large number of unresolved residues in the absence of a bound guide RNA (43, 70). Upon comparison to Cas9 RNP or ternary structures, these regions become structured and appear to be heavily involved in RNA binding (5, 43, 48, 70). In addition, the binding of crRNA and *trans*-acting crRNA guides, as well as single-guide RNAs, to Cas9 have been proposed to modulate the conformational dynamics of Cas9 (42, 45, 46).

While our study cannot definitively distinguish between induced fit and conformational selection binding modes during RNP assembly, results are better explained by an induced fit model. In induced fit mechanisms, the ligand binds an “inactive” state of the receptor, usually “loosely” or at low affinity, but then small, sequential conformational changes can occur where the protein creates more ligand contacts. For assembly of RNP complexes, this usually involves induced fit of the RNA rather than the protein or else a mutual induced fit of both protein and RNA (9, 11, 71, 72). Induced fit can be considered a special case of conformational selection but the two are often viewed as competing models (73, 74). Conformational selection posits that the receptor or enzyme occupies all possible states at different likelihoods and toggles between sampling “active” and “inactive” states. Thus, the kinetics of sampling can become important. Only when the protein transiently samples the active state can the ligand bind, which shifts the population of enzyme toward an active form. This creates a “selection” for the active conformation (74).

Some criteria for distinguishing induced fit and conformational selection can be applied (73). First, the range of conformational states can be informative (74, 75). For AsCas12a, the residues and domains that interact with the 5′ handle appear to occupy a broad range of dynamic conformations. This would suggest the ability to accommodate many incremental changes that could tune its structure to fit the crRNA ligand (74, 76). Indeed, the protein appears to wrap around and protect the RNA after complex formation, which generates very low nanomolar binding affinity (77) (Fig. 4). Importantly, however, interdomain angle sampling from MD simulations revealed that the angles, and therefore conformational states, primarily occupied by the RNP form of AsCas12a are almost never sampled in simulations of the apo-AsCas12a. These results strongly argue against conformational selection as a likely mechanism of RNP assembly (63). Previous simulations of U1A protein binding to the hairpin II of U1 snRNA argued for induced fit based on the absence of the bound conformer when not assembled into the RNP. Only when the RNP complex was formed was the bound conformer observed and stable (75), similar to our interdomain angle analyses (Fig. 4, *E* and *F*).

Second, the timescale of conformational transitions, and therefore kinetics, is important (73, 76). If conformational transitions from an unbound/inactive to bound/active structure are infrequent and slow for Cas12a, then conformational selection could be argued. However, AsCas12a transitions in and out of the free and RNP conformational states are very rapid, on a nanosecond timescale, as observed by K15-L807 and H977-D966 residue distance measurements (Fig. 3). Third, although we did not explicitly focus on the role of the variable guide/spacer sequence of crRNA during binding, it is possible that this contributes an additional binding module. The necessity of distinct binding steps to produce high affinity binding of U1A to U1 hairpin II RNA has been used to support induced fit mechanisms (78). The first step was electrostatic-mediated binding, referred to as the “lure,” which could be partially fulfilled by the negatively charged phosphate backbone of the crRNA spacer sequence, followed by a more specific “lock” step, potentially fulfilled by the crRNA 5′ pseudoknot handle. The observation that modified 5′ extensions to the end of the Cas12a crRNA can improve enzyme activity may be partially attributable to improved binding affinity *via* electrostatic interactions with Cas12a (77, 79, 80). Thus, the entire crRNA may participate in stepwise conformational changes that would be indicative of induced fit binding.

In this study, we were limited by the number of Cas12a and crRNA sequences suitable for our analysis. Nonetheless, they were sufficient to generate phylogenetic trees that could assess relatedness and for interpretation of sequence conservation across species. A greater number of sequences could refine our analyses but are unlikely to change the overarching observation of relatively low protein sequence homology compared to high repeat sequence (5′ pseudoknot) conservation. For structural comparisons, we were also limited to the three available structures of Cas12a. However, these were fortuitously well-distributed across the inferred phylogenetic tree, which provided higher confidence that they were representative. Importantly, despite sequence divergence, the final folded structures of Cas12a proteins, especially around the guide RNA 5′ pseudoknot, were highly similar. The crRNA pseudoknot structures themselves were nearly identical. While this study focused more on AsCas12a, simulations using FnCas12a and LbCas12a were also in agreement with our conclusions. Initial model selection can also be a limitation. Our simulations began with manually generated apo-AsCas12a from the DNA-bound ternary structure using coordinate extraction. Beginning with an apo crystal structure could generate fine variations in the results, though we would not expect these to significantly alter the major conclusions from this study. Indeed, beginning with an alternative *de novo* predicted apo-AsCas12a structure from the AlphaFold database resulted in remarkably similar outcomes in our MD simulations.

We also limited our in-solution experimental studies to AsCas12a as a model system. It is likely that limited trypsin proteolysis of other Cas12a members would yield alternative cleavage patterns as these will depend on surface accessibility of lysine or arginine residues. However, we would still expect to observe altered peptide cleavage patterns. Finer mapping of AsCas12a may also be achieved with other peptide hydrolysis methods or proteases and would be expected to create a more complete picture of conformational changes during RNP assembly. Ultimately, other methodologies to map the conformational states of Cas12a and their occupancy level when free and bound to guide RNA, especially at very fast time scales during RNP assembly, would help further establish the molecular mode of binding. These could include experimental structural, thermodynamic, molecular distance, and kinetic measurements that could further support or refute induced fit mechanisms.

## Experimental procedures

### Cas12a sequence alignment, phylogenetic analysis, and structure alignment

Cas12a sequences were downloaded from UniProt (uniprot.org). Sequences less than 800 amino acids and duplicate taxa were removed before alignment with ClustalW (81). This alignment was used for maximum likelihood phylogenetic inference in MEGA11 with 500 bootstrap replicates and the JTT model of protein evolution (82). For simplicity, a consensus tree was produced with nodes having a bootstrap value less than 50 collapsed into polytomies. For comparing Cas12a RNP structures, Cas12a RNP or ternary complexes solved for *Lachnospiraceae bacterium* ND2006 (Lb) Cas12a (PDB ID: 5XUS) (51), *F. tularensis* subsp. *novicida* (Fn) Cas12a (PDB ID: 5NG6) (57), and *Acidaminococcus* sp. strain BV3L6 (As) Cas12a (PDB ID: 5B43) (6) were aligned in PyMOL (https://pymol.org/2/) (v1.5.0.5).

### crRNA repeat alignment and secondary structure prediction

Type V repeat sequences were downloaded from CRISPRdb (https://crispr.i2bc.paris-saclay.fr/). These repeats were aligned and their secondary structure predicted with LocARNA (https://rna.informatik.uni-freiburg.de/LocARNA) (58). A consensus sequence for the alignment was generated with a cut-off of 55%. A visualization of the predicted secondary structure from LocARNA with this consensus sequence was constructed with forna (http://rna.tbi.univie.ac.at/forna/) (83).

### MD structure preparation

#### FnCas12a, and LbCas12a

The crystal structure of the Cas12a complex (PDB ID: 5B43) (6) FnCas12a (PDB ID: 5NG6) (57) and LbCas12a (PDB ID: 5ID6) (51) were used for MD studies. The missing residues in the protein were filled by using the SWISS-MODEL (84) webserver. To generate the Cas12a RNP model, the DNA part of the crystal structure was manually removed. Similarly, the protein only structure was generated by removing the RNA part. The coordinates of the pseudoknot sequence were manually extracted and used for the preparation of the free pseudoknot RNA. The apo structure of AsCas12a from AlphaFold database (https://alphafold.ebi.ac.uk/entry/U2UMQ6) has also been utilized for the simulation. The Amber ff14SB (85) force field for protein, the ff99bsc0+χOL3 (86, 87, 88) force field for RNA, and TIP3P (89) model for water have been employed. The system was solvated using a 10 Å rectangular water box and Na^+^/Cl^-^ ions were added to neutralize the complexes in the tleap module of AmberTools 19.

### MD methodology

The MD protocol used in earlier studies by Palermo and co-workers was used with slight modifications for the protein and RNP systems (56). An initial minimization of 10,000 steps (5000 steps of steepest descent along with 5000 steps of conjugate gradient) with a restraint of 300 kcal/mol·Å^2^ on the biomolecules was carried out followed by another 10,000 steps (5000 steps of steepest descent along with 5000 steps of conjugate gradient) without any restraints. The heating was done in four stages where systems were heated up from 0 to 50 K and 50 to 100 K by running two simulations of 50 ps each (NVT) imposing harmonic restraints of 100 kcal/mol·Å^2^ on the protein and RNA. The temperature was then increased to 200 K in 100 ps (NVT) with a smaller restraint of 25 kcal/mol·Å^2^ and to 300 K in 500 ps without any restraints (NPT). One nanosecond equilibration followed by a test production run of 10 ns was carried out (NPT).

The MD simulation of the pseudoknot was carried out starting with 1000 steps of minimization (500 steps of steepest descent), followed by a 20 ps of equilibration with a restraint force of 25 kcal/mol·Å^2^ on the pseudoknot, at a temperature of 100 K (NVT). A further 2500 steps of unrestrained minimization with 1000 steps of steepest descent and 100 ps of unrestrained equilibration using a constant pressure periodic boundary of 1 atm was carried out where the temperature was increased from 100 K to 300 K. Finally, production run for 1 μs was performed on the protein, RNP complex, and pseudoknot at 300 K using the GPU accelerated version of Particle Mesh Ewald Molecular Dynamics of AMBER 18. The time step used in MD simulations was 2 fs and the cut off for long range electrostatic interaction 10 Å. The SHAKE algorithm was applied to the bonds containing hydrogens. Langevin dynamics was used for temperature control (90) and Berendsen barostat (91) was used for pressure control for all the simulations.

### MD simulation analysis

All the analyses were carried out using the CPPTRAJ (92) module of the AmberTools 19 (https://ambermd.org/AmberTools.php), VMD (https://www.ks.uiuc.edu/Research/vmd/) (93), and PyMOL Molecular Graphics System (https://pymol.org/2/), version 2.0 (Schrodinger, LLC). The RMSD of the protein backbone and the RNA backbone were calculated using the first frame of the 1 μs simulation as the reference. All frames were considered for the calculation. RMSF analysis and the distance between the cα atoms of the amino acids were calculated using the CPPTRAJ module considering every frame of the simulation. The 2DRMSD studies were carried out using every 1000th frame of simulation. The command 2drms in CPPTRAJ was used for the calculation and only the C, N, O, CA, and CB backbone atoms were considered. The information saved was further plotted using gnuplot (http://www.gnuplot.info/). The surf command in CPPTRAJ was used to calculate the SASA, which uses the LCPO algorithm (94). The surface electrostatic potential calculation was carried out using the ABPS electrostatic plugin in PyMOL. This calculation was carried out using the major cluster snapshots of the systems. The trajectories were visualized in VMD and the images were rendered using PyMOL.

### PC analysis

PC analysis was carried out using the whole 1 μs simulation of the MD trajectories. Only the Cα atoms were considered for the analysis. Initially, the translational and rotational motion of the protein is removed by fitting the trajectory to the average MD structure. Further coordinate covariance matrix, eigen values, and eigen vectors were calculated. A pseudo trajectory to visualize the first PC was also generated. The principal motions were visualized using the normal mode wizard plugin in the VMD software.

### Cluster analysis

Average-linked hierarchical agglomerative clustering algorithm was used for the cluster analysis using CPPTRAJ. The rms distances of the protein backbone was used as the reference distance for the clustering procedure. The centroid of the cluster summary was extracted as the major cluster snapshot. The epsilon value for the analysis was 3 Å and every 15th frame was considered for the analysis.

### Limited trypsin hydrolysis and MS of AsCas12a protein and RNP

Plasmid encoding His(6x)-MBP-AsCas12a was obtained from Addgene (79,007) (95) and protein prepared as described (39). crRNA targeting an EGFP sequence (crEGIP) (39) was synthesized and purified by Integrated DNA Technologies. Trypsin proteolysis was performed similarly as described (39). Briefly, 30 μg of AsCas12a was incubated in the presence and absence of crRNA (1:1.5 M ratio) at 23 °C for 5 min. The resulting samples were incubated with Trypsin-EDTA solution (0.05%, Invitrogen) at a mass ratio of 100:1 and incubated at 37 °C for 15 min. The reaction was stopped by the addition of SDS-PAGE loading buffer (New England Biolabs) and incubation at 95 °C for 5 min. The reaction products were analyzed by 12% SDS-PAGE and stained with Coomassie brilliant blue G-250 in 50% (v/v) methanol and 10% (v/v) acetic acid then destained in the same solution without dye. Differential bands of interest were gel extracted and shipped to the UT Southwestern Medical Center Proteomics Core facility for LC/MS/MS mass spectrometric identification using their Gel Band ID service. Data was analyzed using Proteome Discoverer 2.4 (https://www.thermofisher.com/us/en/home/industrial/mass-spectrometry/liquid-chromatography-mass-spectrometry-lc-ms/lc-ms-software/multi-omics-data-analysis/proteome-discoverer-software.html) and searched using the His(6x)-MBP-AsCas12a protein sequence.

### Differential scanning fluorimetry of AsCas12a

Thermal-shift assays were performed using a Bio-Rad CFX96 instrument with the FRET channel. AsCas12a alone and AsCas12a:crRNA (1:1) complex were incubated at room temperature for 10 min in a final assay concentration of 1x DSF buffer (100 mM Hepes, pH 7.5, 100 mM NaCl, 1 mM MgCl_2_). SYPRO Orange Protein Gel Stain 5000× (Thermo Fisher Scientific) was freshly diluted to 171.25× in nuclease-free water and 2 μl was spotted into the wells of a Hard-Shell 96-well PCR Plate (Bio-Rad). The incubated solution was then added to wells to a final assay volume of 25 μl and AsCas12a or AsCas12a:crRNA (1:1) concentration of 3.3 μΜ. The plate was sealed with Microseal “B” PCR Plate Sealing Film (Bio-Rad) and samples were heated from 25 °C to 85 °C at a ramp rate of 1 °C/min. A single fluorescence was collected every 30 s. The melting temperature (*T*_m_) was determined using wTSA-CRAFT (https://bioserv.cbs.cnrs.fr/TSA_CRAFT/) (96).

Temperature-dependent SYPRO Orange emission intensity curves were analyzed to extract midpoint temperatures (*T*_m_) and Van’t Hoff enthalpies of folding (ΔH) using two-state (F ⇌ U) and sequential three-state (F ⇌ I ⇌ U) unfolding models as described (97, 98). Briefly total signals (*S*_t_) in DSF curves were baseline corrected by extrapolating linear regions (99) (*e.g.*, 25–30 °C and 75–80 °C) and fractional signals were fit to each model using unconstrained nonlinear regressions in MATLAB (MathWorks). For each complex, mean values and 95% confidence intervals from three independent trials are reported.

### Nucleic acid thermal denaturation monitored by UV absorbance

Experiments were performed on a Varian Cary 100 Bio UV-Visible Spectrophotometer equipped with a Cary Temperature Controller using a 1 cm path length cuvette. Oligonucleotides were purchased from Integrated DNA Technologies. Samples were prepared at a concentration of 2 μM oligonucleotide in 10 mM sodium phosphate buffer, pH 7.0, and 2 mM MgCl_2_ with a final sample volume of 500 μl. Samples were slow-annealed by heating to 95 °C for 10 min, slowly cooling to 25 °C over 3 h, and then incubating them at 4 °C for 16 h. Absorbance values were acquired at 260 nm over the range of 5 to 95 °C at a ramp rate of 0.5 °C/min with a data collection interval of 0.5 °C. The background was subtracted using Varian software and measurements performed in triplicate. The maximum value of the first derivative curves were used to determine the *T*_m_ values. The average *T*_m_ of each oligonucleotide with the corresponding SD is reported along with the normalized absorbance spectra.

### Nucleic acid CD

Experiments were performed on a Chirascan VX Spectrophotometer using a 1 mm path length cuvette. Oligonucleotide samples were prepared at a concentration of 20 μM with a final sample volume of 200 μl. The buffer and slow-annealing conditions were the same as for thermal denaturation experiments. Ellipticity spectra were recorded over the range of 320 to 200 nm with a bandwidth of 1 nm, a sampling rate of 0.5 s-per-point, and a data collection interval of 1 nm. Three acquisitions were obtained for each oligonucleotide to create an average spectrum, from which the background spectrum was subtracted. The final reported spectra were smoothed using Chirascan software.

## Data availability

The datasets and computer code generated or used in this study are available upon request to P. I. P. Phylogenetic reconstruction datasets and sequences, as well as original MS data, are available upon request to K. T. G.

## Supporting information

This article contains supporting information.

## Conflict of interest

The authors declare that they have no conflicts of interest with the contents of this article.
