# Supplementary material for: Binding to the conserved and stably folded guide RNA pseudoknot induces Cas12a conformational changes during ribonucleoprotein assembly
Source: J Biol Chem. 2023 Apr 12;299(5):104700. doi: 10.1016/j.jbc.2023.104700 (PMC10200996; doi:10.1016/j.jbc.2023.104700)
Supplement: Supporting Figures S1–S23 [file mmc1.pdf]

SUPPORTING INFORMATION

Binding to the conserved and stably folded guide RNA pseudoknot induces Cas12a conformational changes during ribonucleoprotein assembly

Sruthi Sudhakar[1,†], Christopher L. Barkau[2,†], Ramadevi Chilamkurthy[2], Halle M. Barber[3], Adrian A. Pater[4], Sean D. Moran[4], Masad J. Damha[3], P.I. Pradeepkumar[1,*], and Keith T. Gagnon[2,4,*]

[1] Department of Chemistry, Indian Institute of Technology Bombay, Mumbai, 400076, India.
[2] Department of Biochemistry and Molecular Biology, Southern Illinois University, School of Medicine, Carbondale, Illinois, 62901, USA.
[3] Department of Chemistry, McGill University, 801 Sherbrooke St. West, Montreal, QC H3A 0B8, Canada
[4] Department of Chemistry and Biochemistry, Southern Illinois University, Carbondale, Illinois, 62901, USA.
[†] Equally contributing authors.
*Corresponding Authors: ktgagnon@siu.edu, pradeep@chem.iitb.ac.in

```
                         10        20        30        40        50        60        70        80
Lachnospiraceae/1-1228   AASKLEKFTNCYSLSKTLRFKAIPVGKTQENIDNKRLLVEDEKRAEDYKGVKKLLDRYYLSFINDVLHSIKLKN--LNNY
Francisella/1-1300       -MSIYQEFVNKYSLSKTLRFELIPQGKTLENIKARGLILDDEKRAKDYKKAKQIIDKYHQFFIEEILSSVCISEDLLQNY
Acidaminococcus/1-1307   -MTQFEGFTNLYQVSKTLRFELIPQGKTLKHIQEQGFIEEDKARNDHYKELKPIIDRIYKTYADQCLQLVQLDWENLSAA

                         90        100       110       120       130       140       150       160
Lachnospiraceae/1-1228   ISLFRKKTRTEKEN--KELENLEINLRKEIAKAFKG-AAGYKSLFKKDIIET----------ILPEAADD----------
Francisella/1-1300       SDVYFKLKKSDDDNLQKDFKSAKDTIKKQISEYIKD-SEKFKNLFNQNLIDAKKGQESDLILWLKQSKDNGIELFKANSD
Acidaminococcus/1-1307   IDSYRKEKTEETRN---ALIEEQATYRNAIHDYFIGRTDNLTDAINKRHAEIYKG--------LFKAELFNGKVLKQLGT

                         170       180       190       200       210       220       230       240
Lachnospiraceae/1-1228   ----KDEIALVNSFNGFTTAFTGFFDNRENMFSEEAKSTSIAFRCINENLTRYISNMDIFEKVD------AIFDKHEVQE
Francisella/1-1300       ITDIDEALEIIKSFKGWTTYFKGFHENRKNVYSSNDIPTSIIYRIVDDNLPKFLENKAKYESLKDKAPEAINYEQIKKDL
Acidaminococcus/1-1307   VTTTEHENALLRSFDKFTTYFSGFYENRKNVFSAEDISTAIPHRIVQDNFPKFKENCHIFTRLITAVP---SLREHFENV

                         250       260       270       280       290       300       310       320
Lachnospiraceae/1-1228   IKEKILNSDYD----------VEDFFEGEFFNFVLTQEGIDVYNAIIGG-FVTESGEKIKGLNEYINLYNAKT-KQALPK
Francisella/1-1300       AEELTFDIDYKTSEVNQRVFSLDEVFEIANFNNYLNQSGITKFNTIIGGKFVNGENTKRKGINEYINLYSQQINDKTLKK
Acidaminococcus/1-1307   KKAIGIFVSTS----------IEEVFSFPFYNQLLTQTQIDLYNQLLGGISREAGTEKIKGLNEVLNLAIQKNDETAHII

                         330       340       350       360       370       380       390       400
Lachnospiraceae/1-1228   FKP------LYKQVLSDRESLSFYGEGYTSDEEVLEVFRNTLN-----KNSEIFSSIKKLEKLFKNFDEYSS--AGIFVK
Francisella/1-1300       YKMSV----LFKQILSDTESKSFVIDKLEDDSDVVTTMQSFYEQIAAFKTVEEKSIKETLSLLFDDLKAQKLDLSKIYFK
Acidaminococcus/1-1307   ASLPHRFIPLFKQILSDRNTLSFILEEFKSDEEVIQSFCKYKT------LLRNENVLETAEALFNELNSIDLT---HIFI

                         410       420       430       440       450       460       470       480
Lachnospiraceae/1-1228   NGPAISTISKDIFGEWNLIR---------DKWNAEYDDIHLKKKAVVTEKYEDDRRKSFKKIGSFSLEQLQEYADADLSV
Francisella/1-1300       NDKSLTDLSQQVFDDYSVIGTAVLEYITQQIAPKNLDNPSKKEQELIAKKTEKAKYLSLETIK-LALEEFNKHRDIDKQC
Acidaminococcus/1-1307   SHKKLETISSALCDHWDTLR-------------------NALYERRISELTGKITKSAKEKVQRSLKHEDINLQEIISAA

                         490       500       510       520       530       540       550       560
Lachnospiraceae/1-1228   VEKLKEIIIQKVDEIYKVYGSS-EKLFDADFVLEKSLKK------NDAVVAIMKDLLDSVKSFENYIKAFFGEGKE----
Francisella/1-1300       RFEEILANFAAIPMIFDEIAQNKDNLAQISIKYQNQGKKDLLQASAEDDVKAIKDLLDQTNNLLHKLKIFHISQSEDKAN
Acidaminococcus/1-1307   GKELSEAFKQKTSEILSHAHAALDQPLPTTLKKQ-------------EEKEILKSQLDSLLGLYHLLDWFAVDESN----

                         570       580       590       600       610       620       630       640
Lachnospiraceae/1-1228   -TNRDESFYGDFVLAYDILLKVDHIYDAIRNYVTQKPYSKDKFKLYFQNPQFMGGWDKDKETDYRATILRYGSKYYLAIM
Francisella/1-1300       ILDKDEHFYLVFEECYFELANIVPLYNKIRNYITQKPYSDEKFKLNFENSTLANGWDKNKEPDNTAILFIKDDKYYLGVM
Acidaminococcus/1-1307   --EVDPEFSARLTGIKLEMEPSLSFYNKARNYATKKPYSVEKFKLNFQMPTLASGWDVNKEKNNGAILFVKNGLYYLGIM
```

```
                                650       660       670       680       690       700       710       720
Lachnospiraceae/1-1228      DK-KYAKCLQKIDKDDVNGN-YEKINYKLLPGPNKMLPKVFFSKKWMAYYNPSEDIQKIYKNGTFKKGD-----------
Francisella/1-1300          NK-KNNKIFDDKAIKENKGEGYKKIVYKLLPGANKMLPKVFFSAKSIKFYNPSEDILRIRNHSTHTKNGSPQKGYEK---
Acidaminococcus/1-1307      PKQKGRYKALSFEPTEKTSEGFDKMYYDYFPDAAKMIPKCSTQLKAVTAHFQTHTTPILLSNNFIEPLEITKEIYDLNNP

                                730       740       750       760       770       780       790       800
Lachnospiraceae/1-1228      ------------------MFNLNDCHKLIDFFKDSISRYPKWSNAYDFNFSETEKYKDIAGFYREVEEQGYKVSFESASK
Francisella/1-1300          -----------------FEFNIEDCRKFIDFYKQSISKHPEWK-DFGFRFSDTQRYNSIDEFYREVENQGYKLTFENISE
Acidaminococcus/1-1307      EKEPKKFQTAYAKKTGDQKGYREALCKWIDFTRDFLSKYTKTTSIDLSSLRPSSQYKDLGEYYAELNPLLYHISFQRIAE

                                810       820       830       840       850       860       870       880
Lachnospiraceae/1-1228      KEVDKLVEEGKLYMFQIYNKDFSDKSHGTPNLHTMYFKLLFDENNHGQIR--LSGGAELFMRRASLKKEELVVHPANSPI
Francisella/1-1300          SYIDSVVNQGKLYLFQIYNKDFSAYSKGRPNLHTLYWKALFDERNLQDVVYKLNGEAELFYRKQSIPKK--ITHPAKEAI
Acidaminococcus/1-1307      KEIMDAVETGKLYLFQIYNKDFAKGHHGKPNLHTLYWTGLFSPENLAKTSIKLNGQAELFYRPKSRMKR--MAHRLGEKM

                                890       900       910       920       930       940       950       960
Lachnospiraceae/1-1228      ANKNPDNPKK----------------------------------TTTLSYDVYKDKRFSEDQYELHIPIAINKCPKN-IF
Francisella/1-1300          ANKNKDNPKK----------------------------------ESVFEYDLIKDKRFTEDKFFFHCPITINFKSSG-AN
Acidaminococcus/1-1307      LNKKLKDQKTPIPDTLYQELYDYVNHRLSHDLSDEARALLPNVITKEVSHEIIKDRRFTSDKFFFHVPITLNYQAANSPS

                                970       980       990      1000      1010      1020      1030      1040
Lachnospiraceae/1-1228      KINTEVRVLLKHDDN-PYVIGIDRGERNLLYIVVVDGKGNIVEQYSLNEIINNFNGIRIKTDYHSLLDKKEKERFEARQN
Francisella/1-1300          KFNDEINLLLKEKANDVHILSIDRGERHLAYYTLVDGKGNIIKQDTFNIIGND----RMKTNYHDKLAAIEKDRDSARKD
Acidaminococcus/1-1307      KFNQRVNAYLKEHPE-TPIIGIDRGERNLIYITVIDSTGKILEQRSLNTIQQF--------DYQKKLDNREKERVAARQA

                               1050      1060      1070      1080      1090      1100      1110      1120
Lachnospiraceae/1-1228      WTSIENIKELKAGYISQVVHKICELVEKYDAVIALEDLNSGFKNSR-VKVEKQVYQKFEKMLIDKLNYMVDKKSNPCATG
Francisella/1-1300          WKKINNIKEMKEGYLSQVVHEIAKLVIEYNAIVVFEDLNFGFKRGR-FKVEKQVYQKLEKMLIEKLNYLVFKDNEFDKTG
Acidaminococcus/1-1307      WSVVGTIKDLKQGYLSQVIHEIVDLMIHYQAVVVLENLNFGFKSKRTGIAEKAVYQQFEKMLIDKLNCLVLKDYPAEKVG

                               1130      1140      1150      1160      1170      1180      1190      1200
Lachnospiraceae/1-1228      GALKGYQITNKFESFKSMSTQNGFIFYIPAWLTSKIDPSTGFVN-LLKTKYTSIADSKKFISSFDRIMYVPEEDLFEFAL
Francisella/1-1300          GVLRAYQLTAPFETFKKMGKQTGIIYYVPAGFTSKICPVTGFVN-QLYPKYESVSKSQEFFSKFDKICYNLDKGYFEFSF
Acidaminococcus/1-1307      GVLNPYQLTDQFTSFAKMGTQSGFLFYVPAPYTSKIDPLTGFVDPFVWKTIKNHESRKHFLEGFDFLHYDVKTGDFILHF

                               1210      1220      1230      1240      1250      1260      1270      1280
Lachnospiraceae/1-1228      DYK------------------NFSRTDADYIKKWKLYSYGNRIRIFAAAKKNNVFAWEEVCLTSAYKELFNKYGINYQQG
Francisella/1-1300          DYK------------------NFG--DKAAKGKWTIASFGSRLINFRNSDKNHNWDTREVYPTKELEKLLKDYSIEYGHG
Acidaminococcus/1-1307      KMNRNLSFQRGLPGFMPAWDIVFEKNETQFDAKGTPFIAGKRIVPVIENHR-FTGRYRDLYPANELIALLEEKGIVFRDG
```

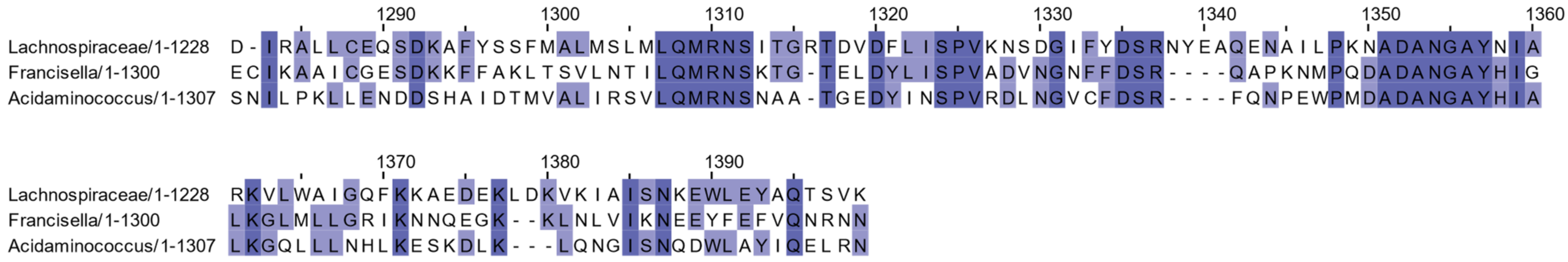

Figure S1. Alignment and conservation of As, Lb, and Fn Cas12a protein sequences. Visualization of sequence alignments and conservation was performed in Jalview. Blue shaded boxes indicate relative degree of sequence conservation.

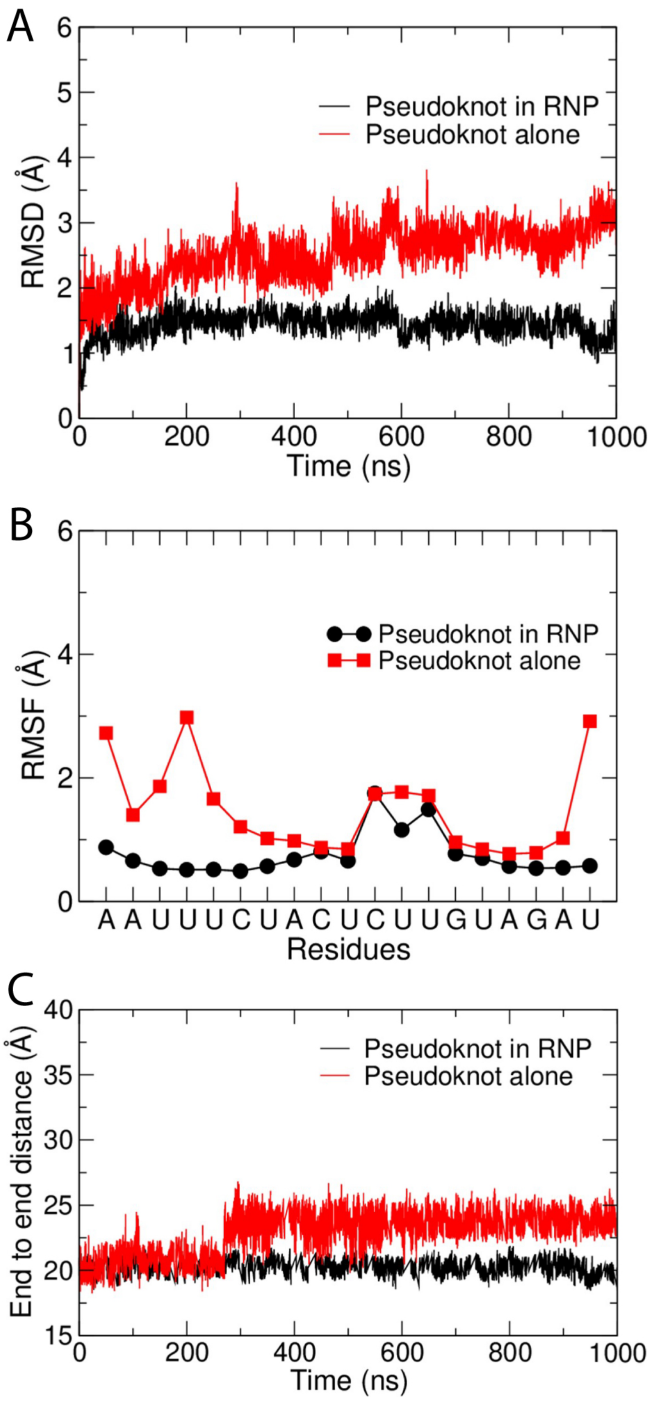

<u>Figure S2</u>. RMSD, RMSF and end-to-end distance graphs of the pseudoknot in free and AsCas12a-bound forms. The free form is represented in red and the pseudoknot bound to RNP complex in black. (**A**) The RMSD values were calculated from the 1 µs trajectories. (**B**) Per nucleotide RMSF of the pseudoknot in the free and bound form. (**C**) End-to end distance of the pseudoknot in the free and bound form. The distances were calculated from the 1 µs trajectories.

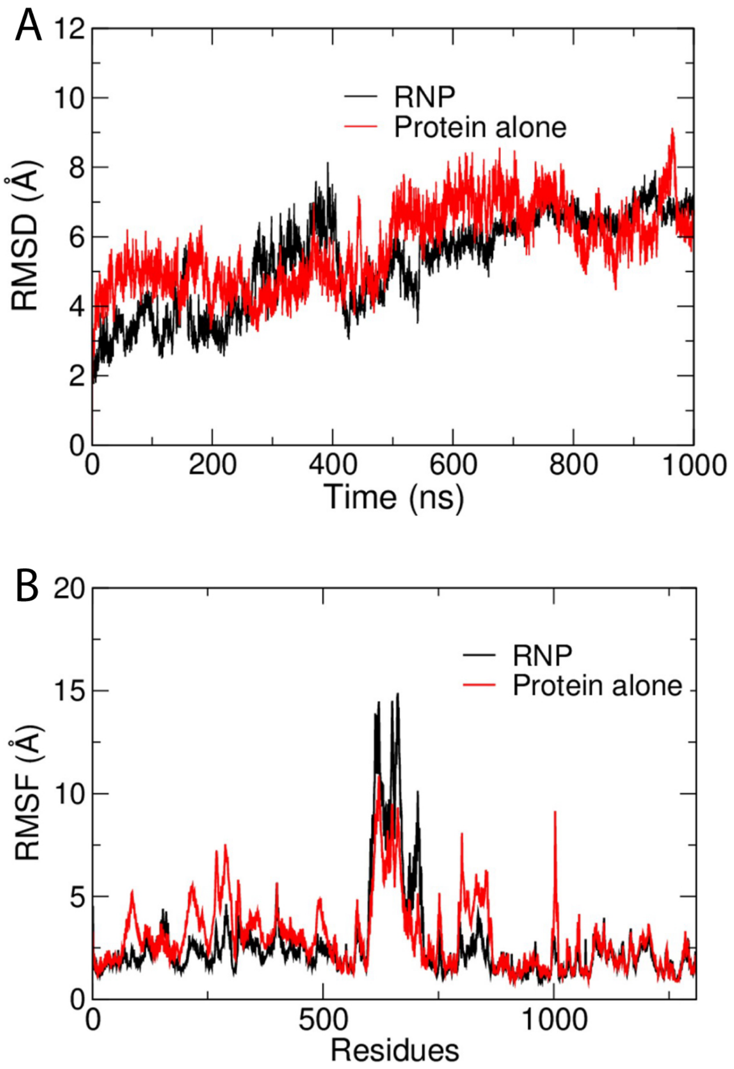

<u>Figure S3</u>. RMSD and RMSF graphs of AsCas12a protein and AsCas12a RNP complex. The free AsCas12a protein is represented in red and the protein in the Cas12a RNP complex is represented in black. (**A**) RMSD graphs of the backbone atoms of protein. RMSD values were calculated from the 1 µs trajectories. (**B**) Per residue RMSF of the AsCas12a protein system and AsCas12a RNP complex. The AsCas12a protein system is represented in red and protein in the RNP complex is represented in black.

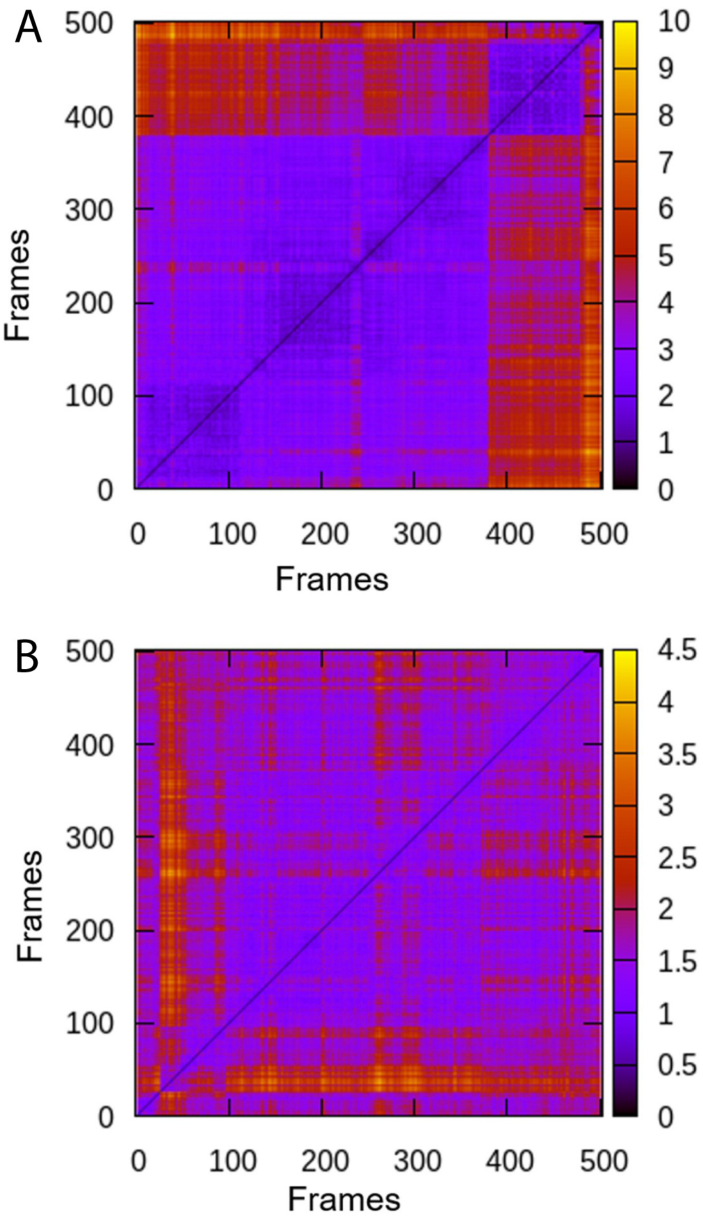

Figure S4. The 2D-RMSD plots of WED-III domain. (**A**) Cas12a protein alone simulation and (**B**) Cas12a RNP complex simulation. The 2D-RMSD values were calculated from every 500th frame of the 1 μs trajectories.

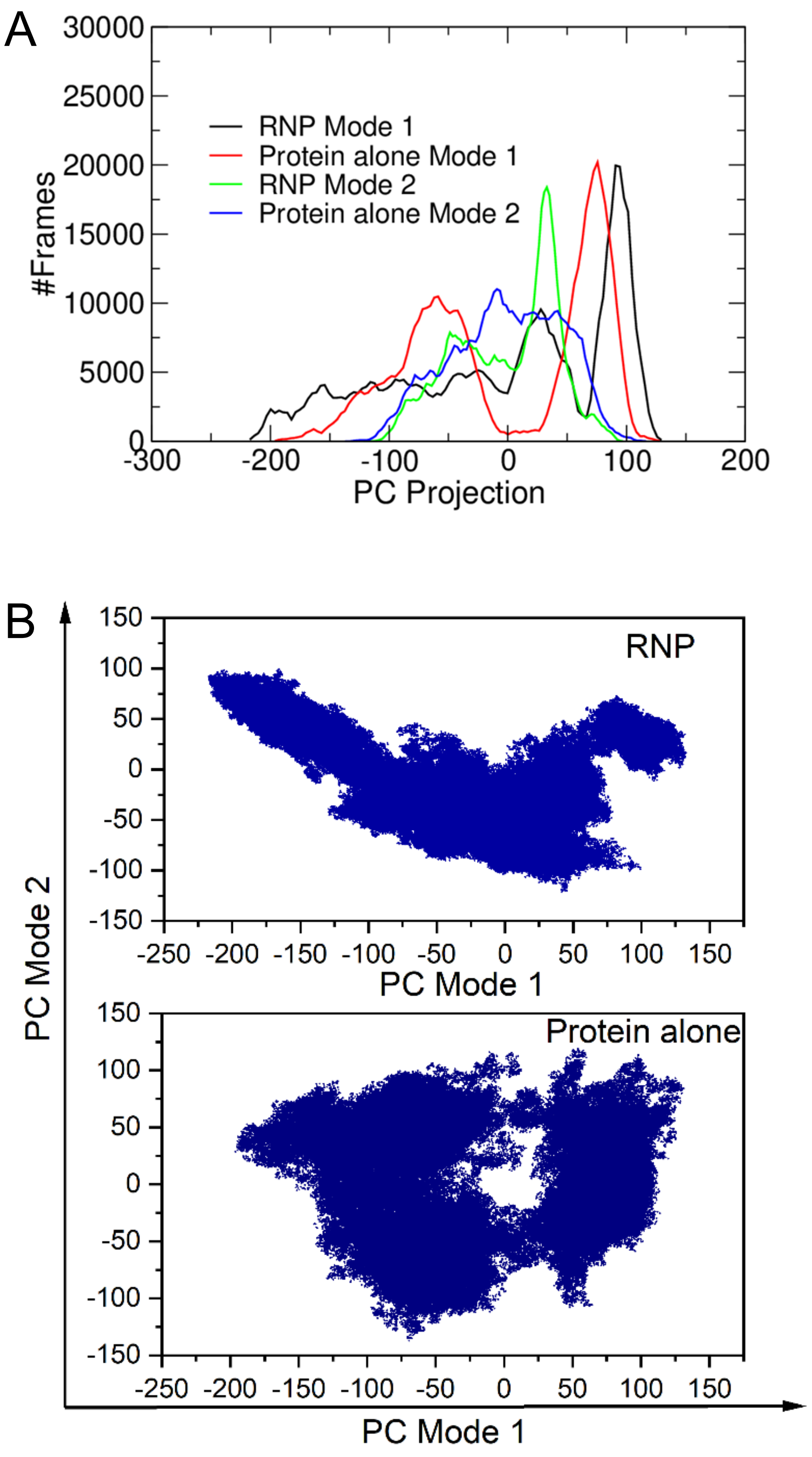

<u>Figure S5</u>. (**A**) The Principal component projection of the top 2 modes of AsCas12a RNP and protein alone systems. (**B**) The scatter plot showing PC mode 1 vs PC mode 2 distribution of AsCas12a RNP and protein alone systems.

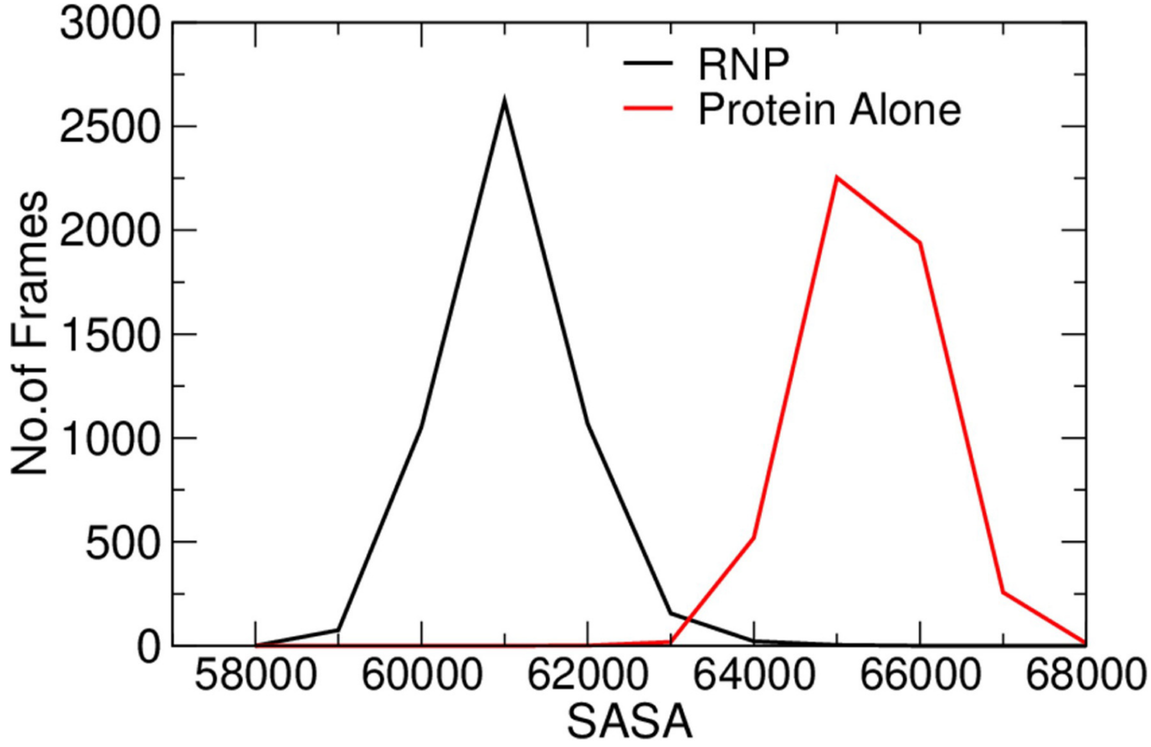

<u>Figure S6</u>. Solvent accessible surface area of the Cas12a protein and Cas12a-crRNA (RNP) complex. The Cas12a protein is represented in red and protein in the Cas12a RNP complex is represented in black.

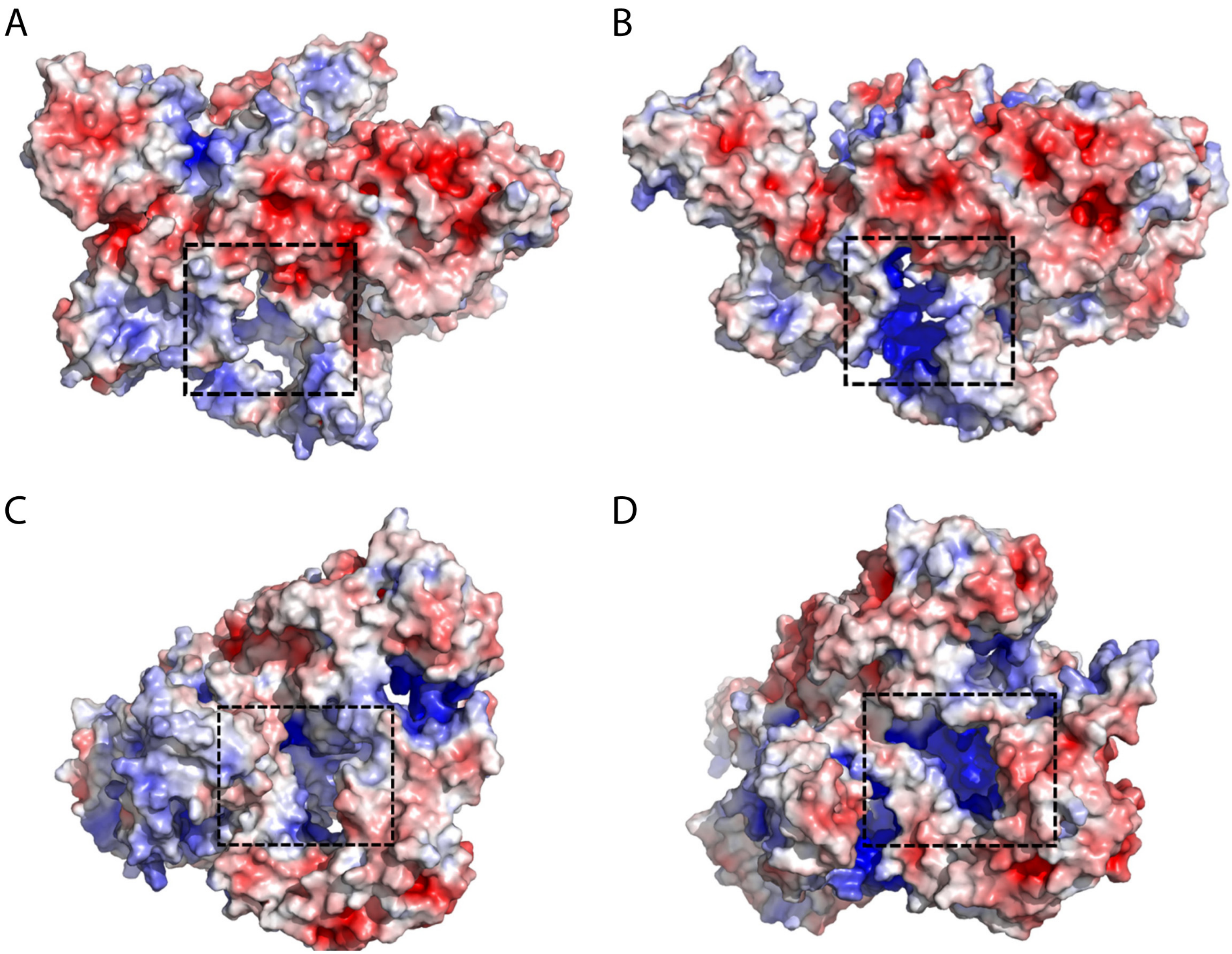

Figure S7. The surface electrostatic potential image of (**A**) pseudoknot occupied region of the Cas12a protein system; (**B**) pseudoknot occupied region of the Cas12a RNP complex; (**C**) ssRNA occupied region of the Cas12a protein system and (**D**) ssRNA occupied region of the Cas12a RNP complex. The positive charged surface is represented in blue and negatively charged surface is represented in red.

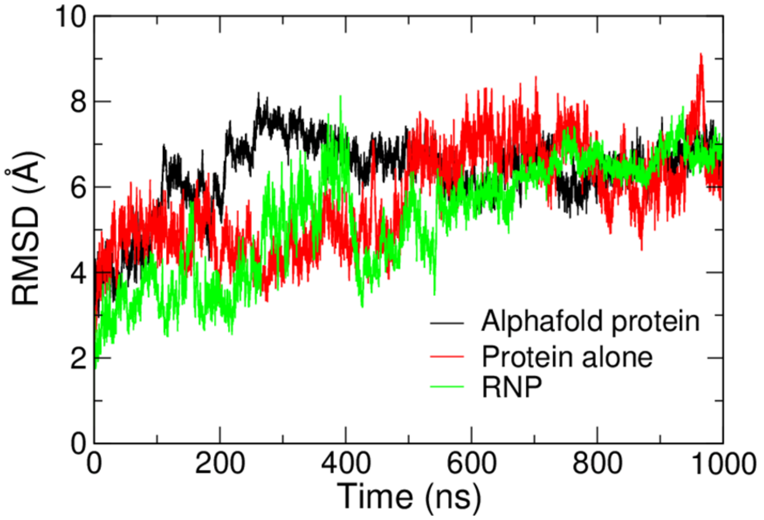

Figure S8. RMSD graphs of the backbone atoms of AsCas12a protein from Alphafold database, protein model generated manually, and AsCas12a RNP complex. The Cas12a protein from the Alphafold database is represented in black, Cas12a generated manually in red, and protein in the Cas12a RNP complex in green. The RMSD values were calculated from the 1 µs MD trajectories.

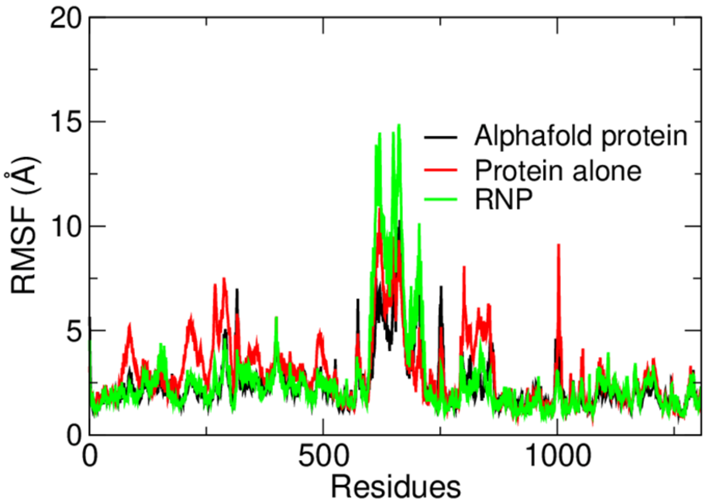

Figure S9. Per residue RMSF of AsCas12a protein from the Alphafold database, protein model generated manually, and AsCas12a RNP complex. The Cas12a protein from the Alphafold database is represented in black, Cas12a generated manually in red, and protein in the Cas12a RNP complex in green.

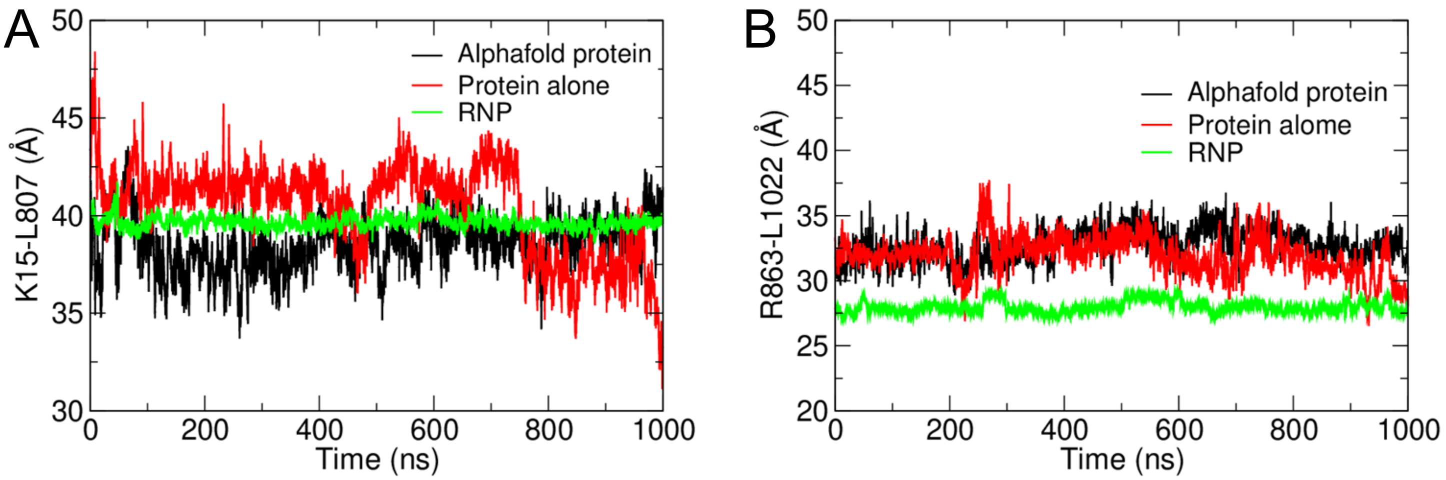

<u>Figure S10</u>. The distance between the Cα atoms of (**A**) K15 and L807; (**B**) R863 and L1022 from the 1 μs MD trajectories. The running averages of the distances are represented in the plot. The AsCas12a protein from the Alphafold database is shown in black, AsCas12a generated manually in red, and protein in the AsCas12a RNP complex in green.

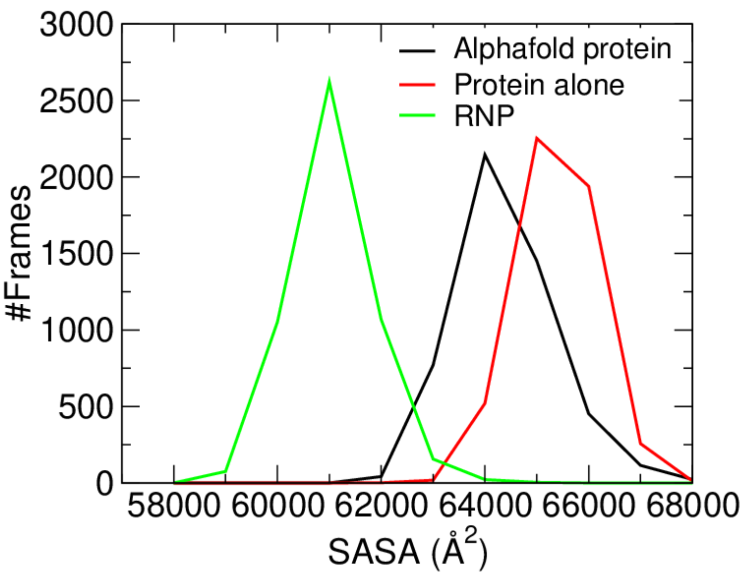

<u>Figure S11</u>. The solvent-accessible surface area of the AsCas12a protein from the Alphafold database, the protein model generated manually, and the AsCas12a RNP complex. The Cas12a protein from the Alphafold database is shown in black, Cas12a generated manually in red, and protein in the Cas12a RNP complex in green.

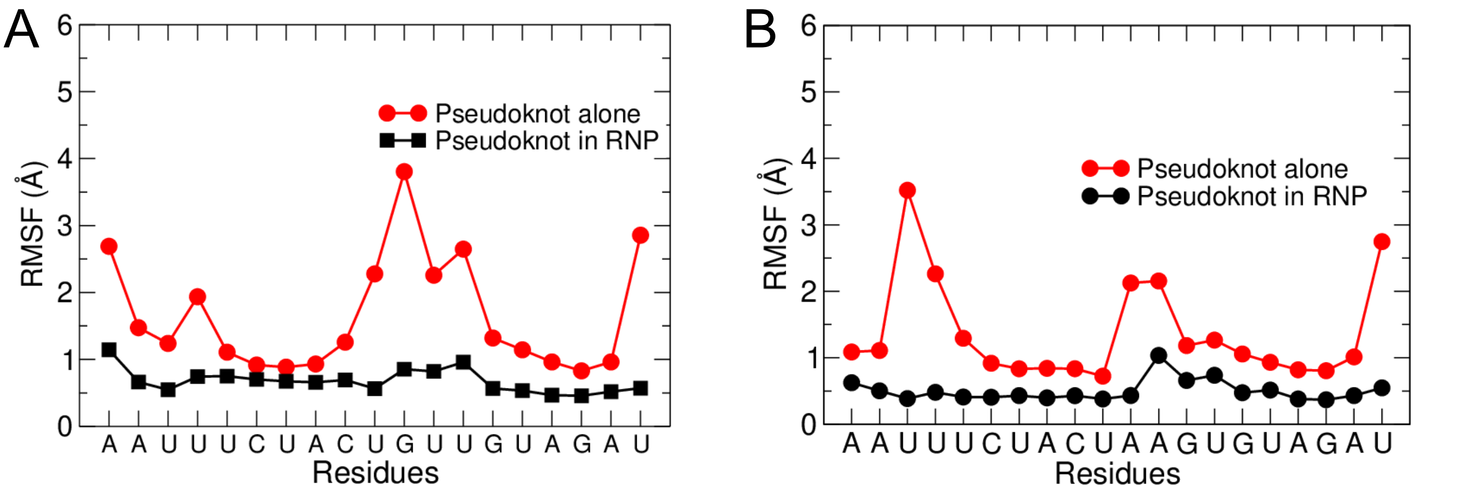

<u>Figure S12</u>. Per nucleotide RMSF of the pseudoknot in the free and bound form of (**A**) FnCas12a; (**B**) LbCas12a. The free form is represented in red, and the pseudoknot bound to the RNP complex in black.

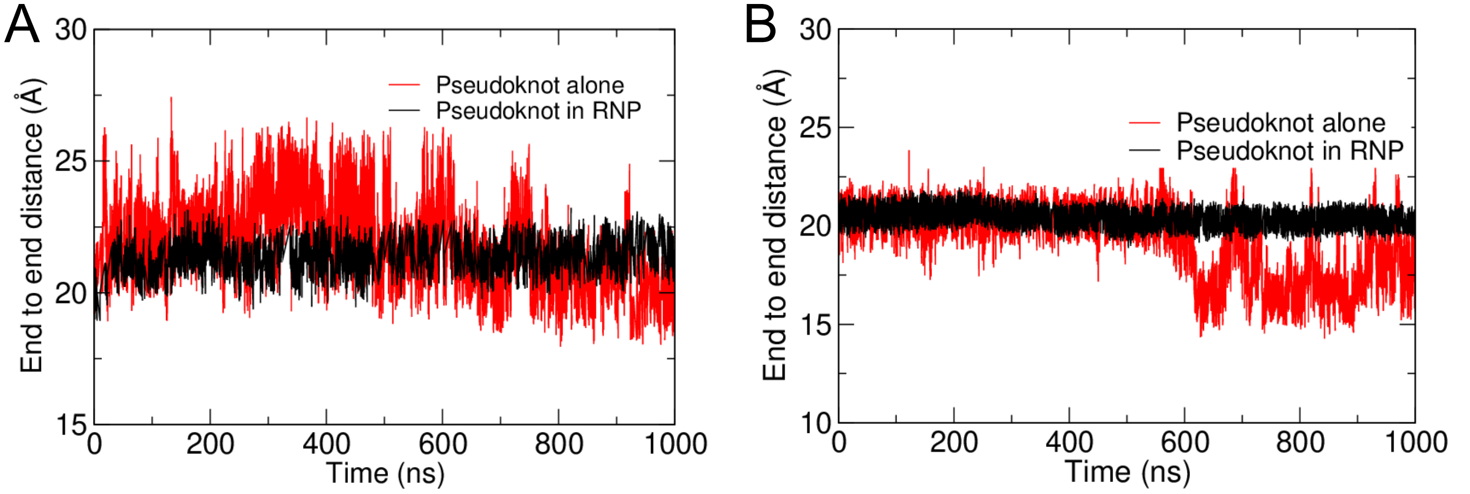

<u>Figure S13</u>. End-to-end distance of the pseudoknot in the free and bound form in (**A**) FnCas12a; (**B**) LbCas12a. The free form is represented in red, and the pseudoknot bound to the RNP complex in black. The distances were calculated from the 1 µs MD trajectories.

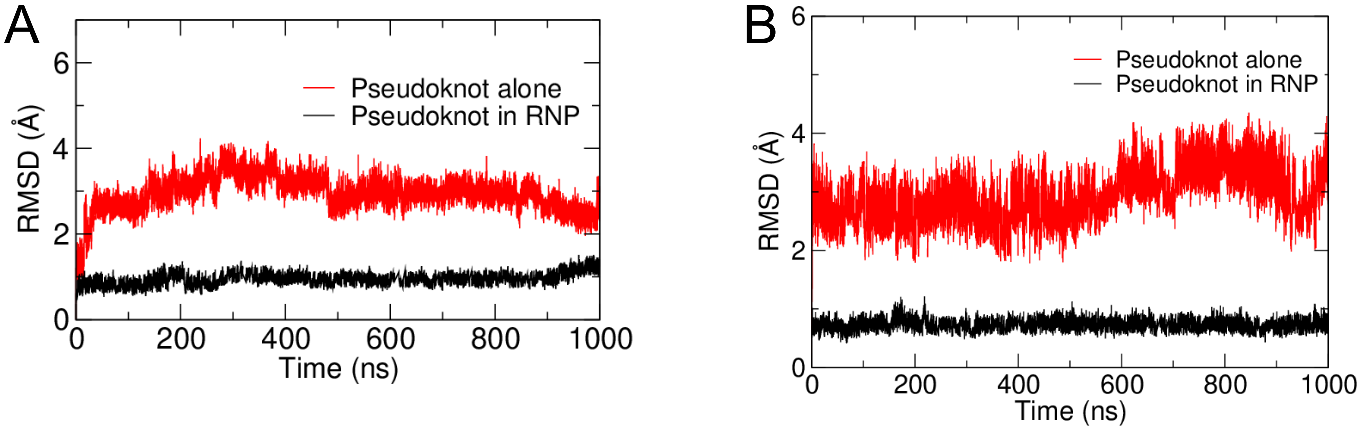

<u>Figure S14</u>. RMSD graphs of the backbone atoms of the free and bound forms of pseudoknot in (**A**) FnCas12a; (**B**) LbCas12a. The free form is represented in red, and the pseudoknot bound to the RNP complex in black. The RMSD values were calculated from the 1 µs MD trajectories.

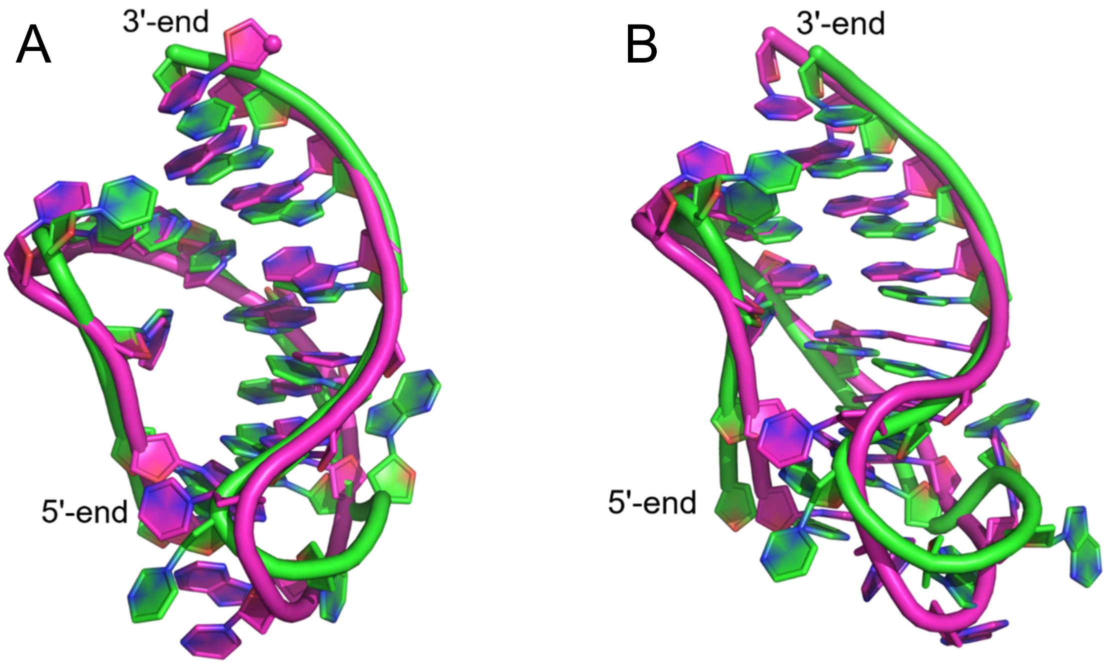

<u>Figure S15</u>. Superimposed image of the representative structures of the major cluster in the free pseudoknot and bound pseudoknot in the RNP complex in (**A**) FnCas12a; (**B**) LbCas12a from the 1 µs MD trajectories. The carbon atoms of the free pseudoknot are represented in magenta, and the carbon atoms of the bound pseudoknot in green, nitrogen atoms in blue and oxygen atoms in red.

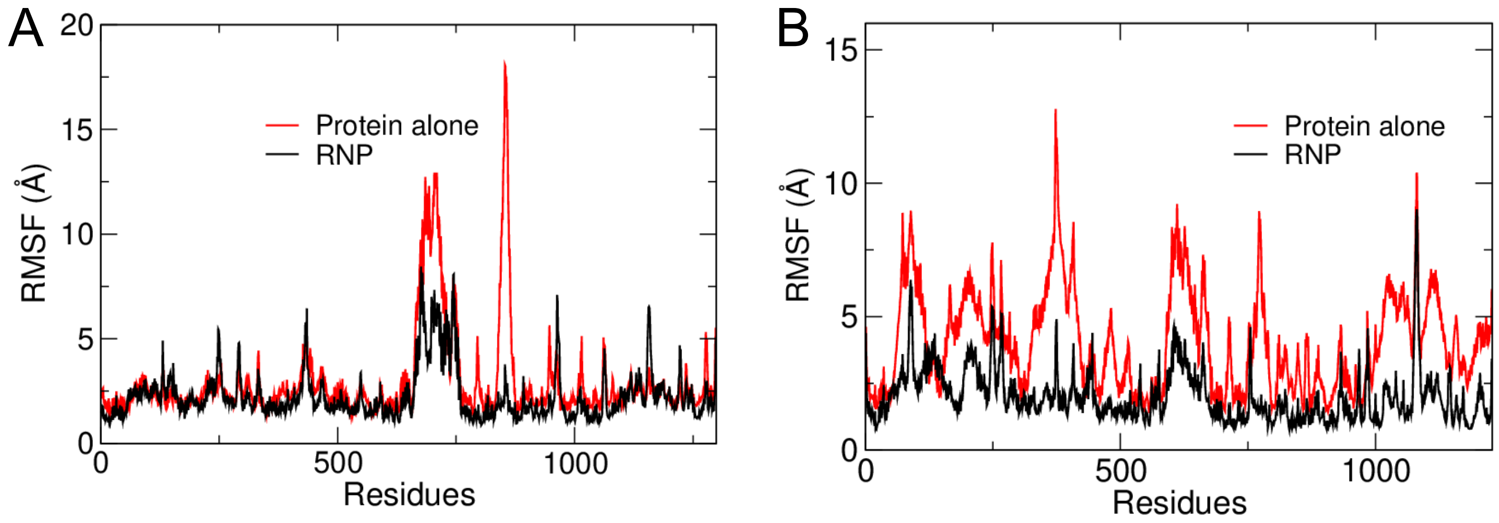

<u>Figure S16</u>. Per residue RMSF of the Cas12a protein system and Cas12a RNP complex in (**A**) FnCas12a; (**B**) LbCas12a. The Cas12a protein system is represented in red, and protein in the Cas12a RNP complex in black. The residues are numbered -1 in FnCas12a and +1 in LbCas12a from the actual crystal structure in the RMSF plots.

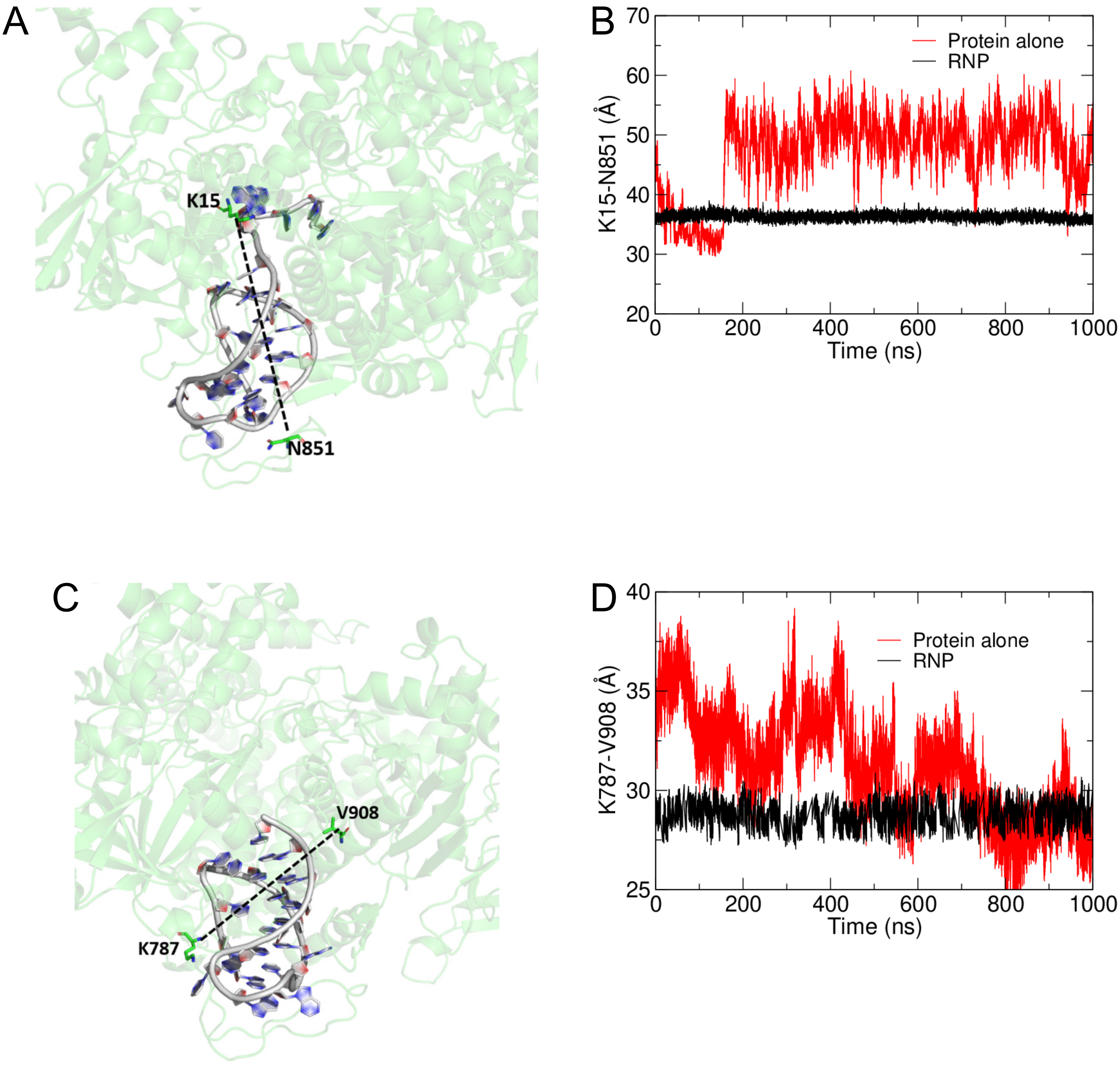

<u>Figure S17</u>. (**A**) Representative image illustrating the amino acids considered for the distance measurements in FnCas12a. (**B**) The distance between the Cα atoms of K15 and N851 of FnCas12a. (**C**) Representative image illustrating the amino acids considered for the distance measurements in LbCas12a. (**D**) The distance between the Cα atoms of K787 and V908 of LbCas12a. The distances are measured from the 1 μs MD trajectories.

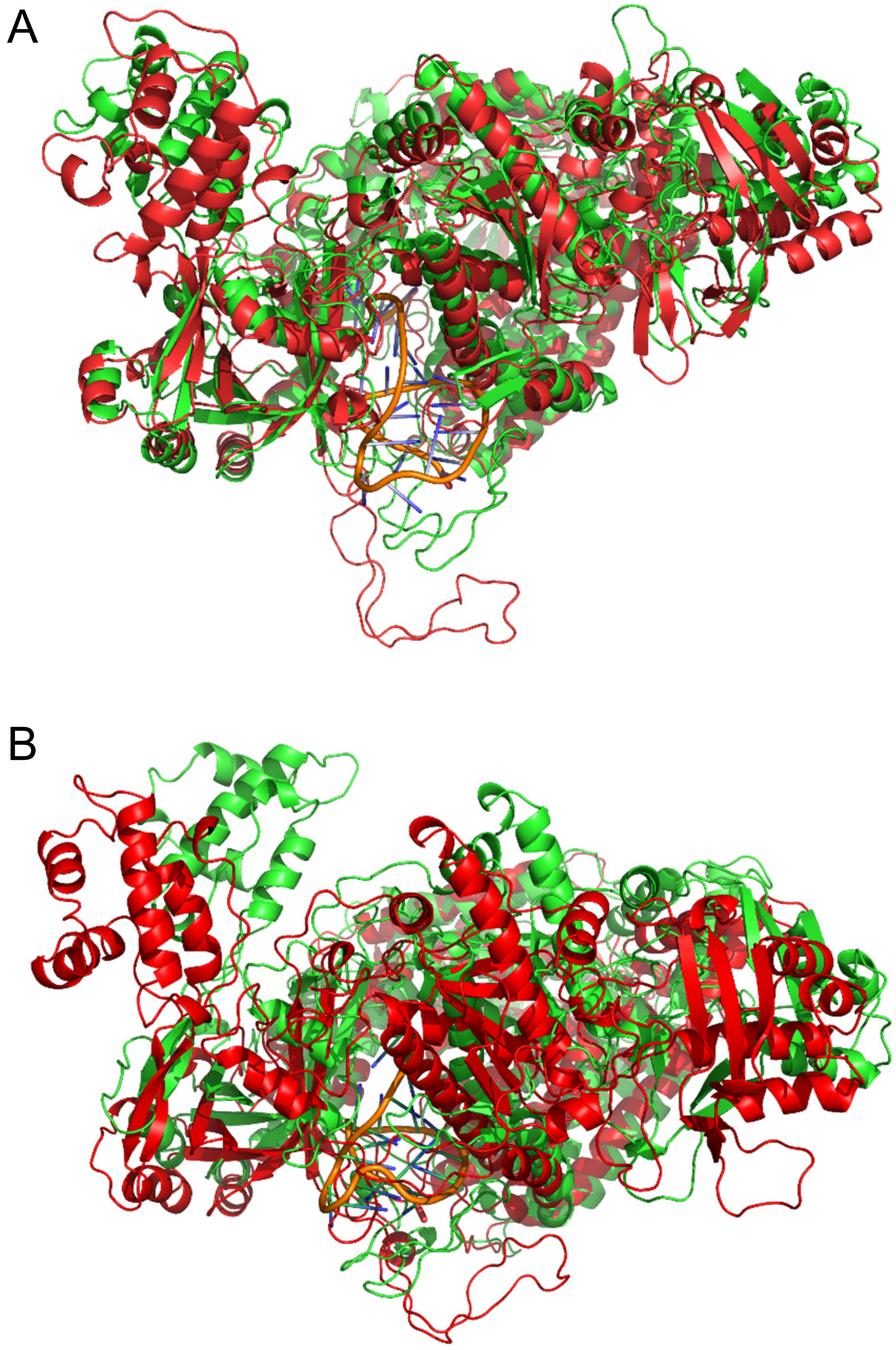

<u>Figure S18</u>. Superimposed images of the major clusters of Cas12a protein and Cas12a RNP complex in (**A**) FnCas12a; (**B**) LbCas12a. The Cas12a protein system is represented in red, and the Cas12a RNP complex in green.

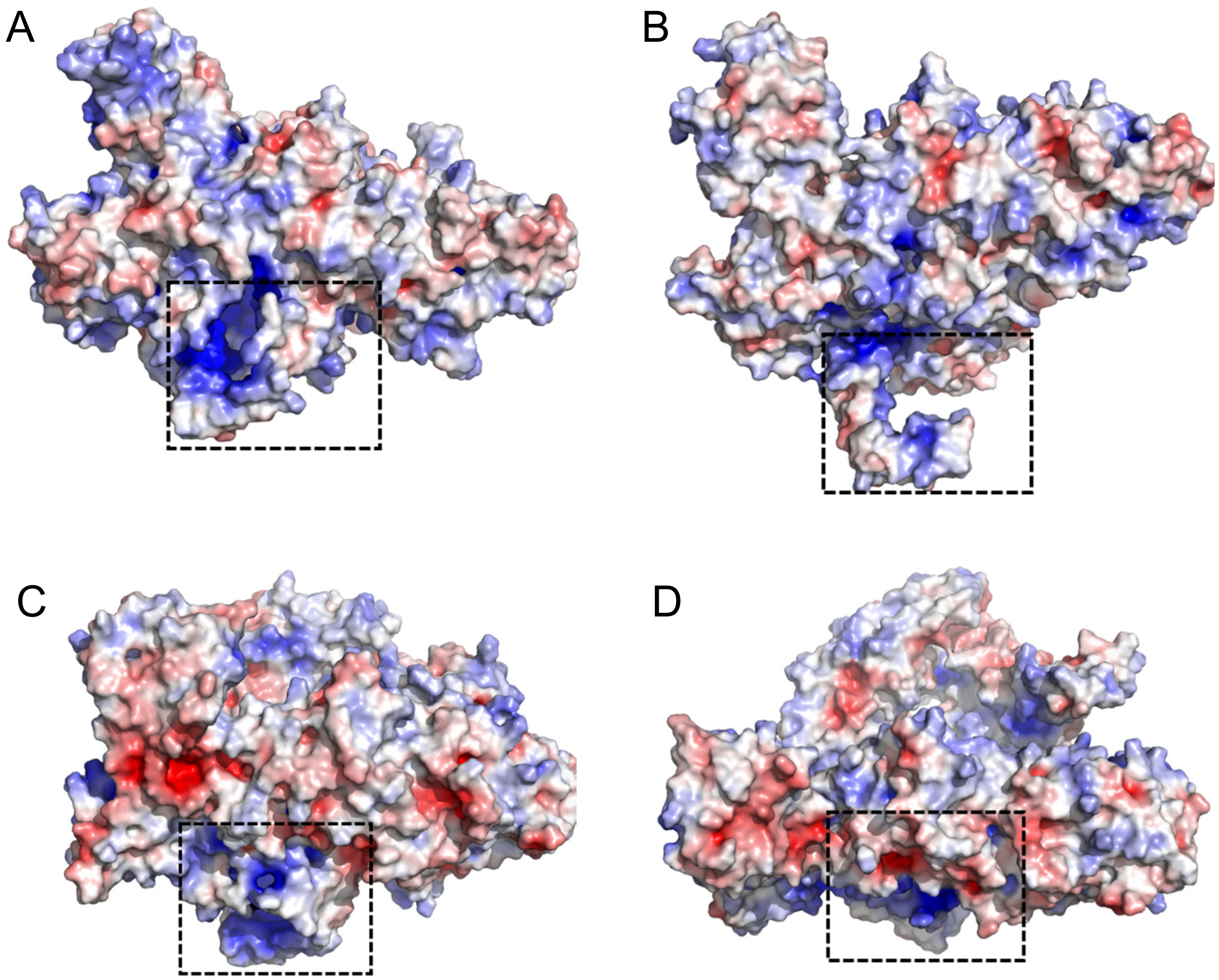

<u>Figure S19</u>. The surface electrostatic potential image of (**A**) pseudoknot-occupied region of the FnCas12a RNP complex; (**B**) pseudoknot-occupied region of the FnCas12a protein alone; (**C**) pseudoknot-occupied region of the LbCas12a RNP complex; (**D**) pseudoknot occupied region of the FnCas12a protein alone. The positively charged surface is represented in blue and negatively charged surface is represented in red. The location of the pseudoknot and point of distinction are marked by dashed rectangular boxes.

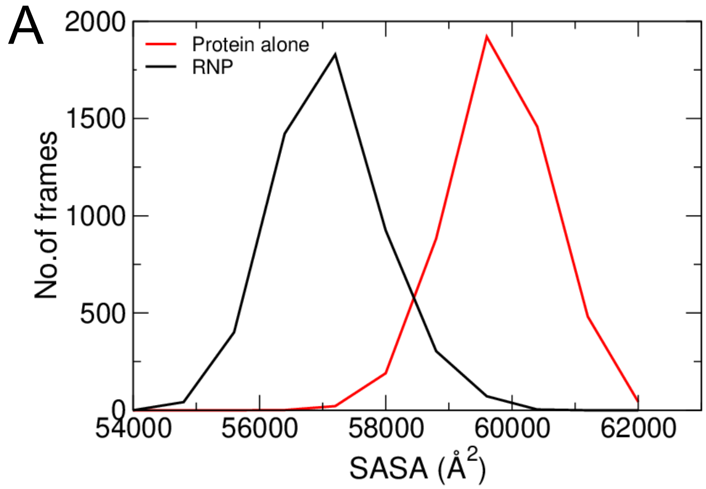

<u>Figure S20</u>. The solvent-accessible surface area of the protein in (**A**) FnCas12a protein system and RNP complex; (**B**) LbCas12a protein system and RNP complex. The Cas12a protein system is represented in red, and protein in the Cas12a RNP complex in black.

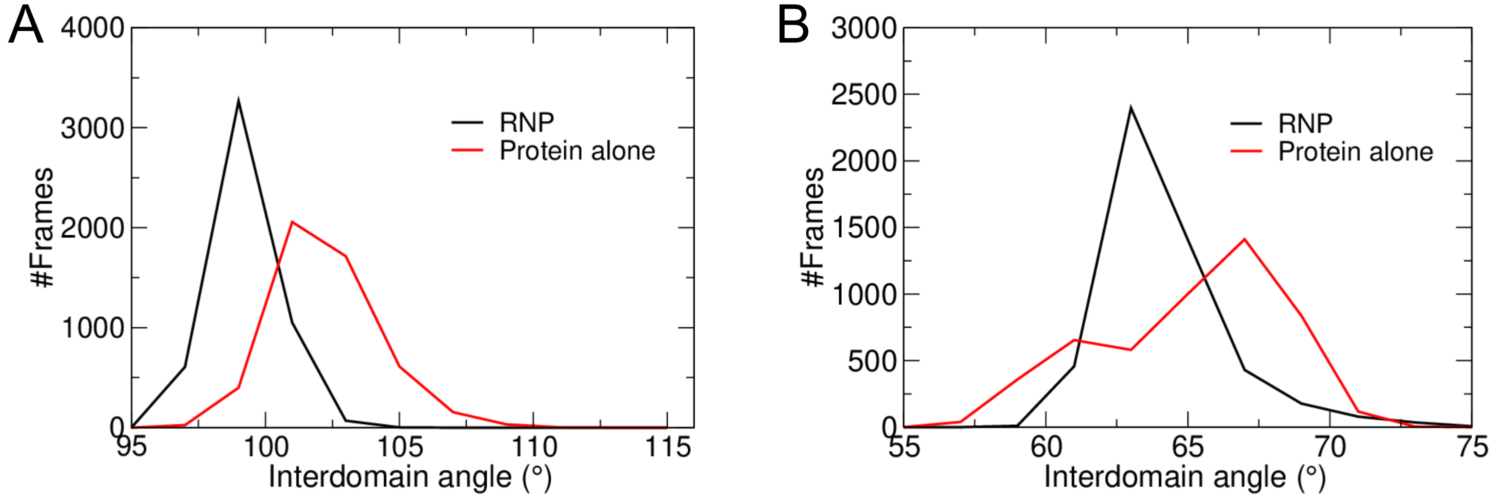

<u>Figure S21</u>. The interdomain angle between (**A**) WED-II: WED-III: RuvC domain; (**B**) REC2: RuvC: NUC domain. The histogram is sampled from the 1 μs MD trajectories. The Cas12a protein alone is shown in red, and the Cas12a RNP in black.

<u>Figure S22</u>. Sequence Analysis of Mass Spec Data for AsCas12a Limited Trypsin Proteolysis

>His6X-MBP-AsCas12a

MKIHHHHHHEEGKLVIWINGDKGYNGLAEVGKKFEKDTGIKVTVEHPDKLEEKFPQVAATGDGPDIIFWAHDRFGGY
AQSGLLAEITPDKAFQDKLYPFTWDAVRYNGKLIAYPIAVEALSLIYNKDLLPNPPKTWEEIPALDKELKAKGKSALMFNL
QEPYFTWPLIAADGGYAFKYENGKYDIKDVGVDNAGAKAGLTFLVDLIKNKHMNADTDYSIAEAAFNKGETAMTINGP
WAWSNIDTSKVNYGVTVLPTFKGQPSKPFVGVLSAGINAASPNKELAKEFLENYLLTDEGLEAVNKDKPLGAVALKSYE
EELAKDPRIAATMENAQKGEIMPNIPQMSAFWYAVRTAVINAASGRQTVDEALKDAQTNSITSLYKKAGFMGAPPKK
KRKVGGMTQFEGFTNLYQVSKTLRFELIPQGKTLKHIQEQGFIEEDKARNDHYKELKPIIDRIYKTYADQCLQLVQLDWE
NLSAAIDSYRKEKTEETRNALIEEQATYRNAIHDYFIGRTDNLTDAINKRHAEIYKGLFKAELFNGKVLKQLGTVTTTEHEN
ALLRSFDKFTTYFSGFYENRKNVFSAEDISTAIPHRIVQDNFPKFKENCHIFTRLITAVPSLREHFENVKKAIGIFVSTSIEEVF
SFPFYNQLLTQTQIDLYNQLLGGISREAGTEKIKGLNEVLNLAIQKNDETAHIIASLPHRFIPLFKQILSDRNTLSFILEEFKSD
EEVIQSFCKYKTLLRNENVLETAEALFNELNSIDLTHIFISHKKLETISSALCDHWDTLRNALYERRISELTGKITKSAKEKVQ
RSLKHEDINLQEIISAAGKELSEAFKQKTSEILSHAHAALDQPLPTTLKKQEEKEILKSQLDSLLGLYHLLDWFAVDESNEVD
PEFSARLTGIKLEMEPSLSFYNKARNYATKKPYSVEKFKLNFQMPTLASGWDVNKEKNNGAILFVKNGLYYLGIMPKQK
GRYKALSFEPTEKTSEGFDKMYYDYFPDAAKMIPKCSTQLKAVTAHFQTHTTPILLSNNFIEPLEITKEIYDLNNPEKEPKK
FQTAYAKKTGDQKGYREALCKWIDFTRDFLSKYTKTTSIDLSSLRPSSQYKDLGEYYAELNPLLYHISFQRIAEKEIMDAVE
TGKLYLFQIYNKDFAKGHHGKPNLHTLYWTGLFSPENLAKTSIKLNGQAELFYRPKSRMKRMAHRLGEKMLNKKLKDQ
KTPIPDTLYQELYDYVNHRLSHDLSDEARALLPNVITKEVSHEIIKDRRFTSDKFFFHVPITLNYQAANSPSKFNQRVNAYL
KEHPETPIIGIDRGERNLIYITVIDSTGKILEQRSLNTIQQFDYQKKLDNREKERVAARQAWSVVGTIKDLKQGYLSQVIHEI
VDLMIHYQAVVVLENLNFGFKSKRTGIAEKAVYQQFEKMLIDKLNCLVLKDYPAEKVGGVLNPYQLTDQFTSFAKMGT
QSGFLFYVPAPYTSKIDPLTGFVDPFVWKTIKNHESRKHFLEGFDFLHYDVKTGDFILHFKMNRNLSFQRGLPGFMPAW
DIVFEKNETQFDAKGTPFIAGKRIVPVIENHRFTGRYRDLYPANELIALLEEKGIVFRDGSNILPKLLENDDSHAIDTMVALI
RSVLQMRNSNAATGEDYINSPVRDLNGVCFDSRFQNPEWPMDADANGAYHIALKGQLLLNHLKESKDLKLQNGISNQ
DWLAYIQELRNPKKKRKVGGYPYDVPDYA

Band 1 (A)

MKIHHHHHHEEGK

LVIWINGDKGYNGLAEVGKKFEKDTGIKVTVEHPDKLEEKFPQVAATGDGPDIIFWAHDRFGGYAQSGLLAEITPDKAF
QDKLYPFTWDAVRYNGKLIAYPIAVEALSLIYNKDLLPNPPKTWEEIPALDKELKAKGKSALMFNLQEPYFTWPLIAADG
GYAFKYENGKYDIKDVGVDNAGAKAGLTFLVDLIKNKHMNADTDYSIAEAAFNKGETAMTINGPWAWSNIDTSKVNY
GVTVLPTFKGQPSKPFVGVLSAGINAASPNKELAKEFLENYLLTDEGLEAVNKDKPLGAVALKSYEEELAKDPRIAATME
NAQKGEIMPNIPQMSAFWYAVRTAVINAASGRQTVDEALKDAQTNSITSLYKKAGFMGAPPKKKRKVGG

MTQFEGFTNLYQVSKTLRFELIPQGKTLKHIQEQGFIEEDKARNDHYKELKPIIDRIYKTYADQCLQLVQLDWENLSAAID
SYRKEKTEETRNALIEEQATYRNAIHDYFIGRTDNLTDAINKRHAEIYKGLFKAELFNGKVLKQLGTVTTTEHENALLRSFD
KFTTYFSGFYENRKNVFSAEDISTAIPHRIVQDNFPKFKENCHIFTRLITAVPSLREHFENVKKAIGIFVSTSIEEVFSFPFYN
QLLTQTQIDLYNQLLGGISREAGTEKIKGLNEVLNLAIQKNDETAHIIASLPHRFIPLFKQILSDRNTLSFILEEFKSDEEVIQS
FCKYKTLLRNENVLETAEALFNELNSIDLTHIFISHKKLETISSALCDHWDTLRNALYERRISELTGKITKSAKEKVQRSLKHE
DINLQEIISAAGKELSEAFKQKTSEILSHAHAALDQPLPTTLKKQEEKEILKSQLDSLLGLYHLLDWFAVDESNEVDPEFSAR
LTGIKLEMEPSLSFYNKARNYATKKPYSVEKFKLNFQMPTLASGWDVNKEKNNGAILFVKNGLYYLGIMPKQKGRYKAL

SFEPTEKTSEGFDKMYYDYFPDAAKMIPKCSTQLKAVTAHFQTHTTPILLSNNFIEPLEITKEIYDLNNPEKEPKKFQTAYA
KKTGDQKGYREALCKWIDFTRDFLSKYTKTTSIDLSSLRPSSQYKDLGEYYAELNPLLYHISFQRIAEKEIMDAVETGK

LYLFQIYNKDFAKGHHGKPNLHTLYWTGLFSPENLAKTSIKLNGQAELFYRPKSRMKRMAHRLGEKMLNKKLKDQKTPI
PDTLYQELYDYVNHRLSHDLSDEARALLPNVITKEVSHEIIKDRRFTSDKFFFHVPITLNYQAANSPSKFNQRVNAYLKEH
PETPIIGIDRGERNLIYITVIDSTGKILEQRSLNTIQQFDYQKKLDNREKERVAARQAWSVVGTIKDLKQGYLSQVIHEIVDL
MIHYQAVVVLENLNFGFKSKRTGIAEKAVYQQFEKMLIDKLNCLVLKDYPAEKVGGVLNPYQLTDQFTSFAKMGTQSG
FLFYVPAPYTSKIDPLTGFVDPFVWKTIKNHESRKHFLEGFDFLHYDVKTGDFILHFKMNRNLSFQRGLPGFMPAWDIVF
EKNETQFDAKGTPFIAGKRIVPVIENHRFTGRYRDLYPANELIALLEEKGIVFRDGSNILPKLLENDDSHAIDTMVALIRSVL
QMRNSNAATGEDYINSPVRDLNGVCFDSRFQNPEWPMDADANGAYHIALKGQLLLNHLKESKDLKLQNGISNQDWL
AYIQELRNPKKKRKVGGYPYDVPDYA

Band 2 (B)

MKIHHHHHHEEGKLVIWINGDKGYNGLAEVGKKFEKDTGIKVTVEHPDKLEEKFPQVAATGDGPDIIFWAHDRFGGY
AQSGLLAEITPDKAFQDKLYPFTWDAVRYNGKLIAYPIAVEALSLIYNKDLLPNPPKTWEEIPALDKELKAKGKSALMFNL
QEPYFTWPLIAADGGYAFKYENGKYDIKDVGVDNAGAKAGLTFLVDLIKNKHMNADTDYSIAEAAFNKGETAMTINGP
WAWSNIDTSKVNYGVTVLPTFKGQPSKPFVGVLSAGINAASPNKELAKEFLENYLLTDEGLEAVNKDKPLGAVALKSYE
EELAKDPRIAATMENAQKGEIMPNIPQMSAFWYAVRTAVINAASGRQTVDEALKDAQTNSITSLYKKAGFMGAPPKK
KRKVGGMTQFEGFTNLYQVSKTLRFELIPQGKTLKHIQEQGFIEEDKARNDHYKELKPIIDRIYKTYADQCLQLVQLDWE
NLSAAIDSYRKEKTEETRNALIEEQATYRNAIHDYFIGRTDNLTDAINKRHAEIYKGLFKAELFNGKVLKQLGTVTTTEHEN
ALLRSFDKFTTYFSGFYENRKNVFSAEDISTAIPHRIVQDNFPKFKENCHIFTRLITAVPSLREHFENVKKAIGIFVSTSIEEVF
SFPFYNQLLTQTQIDLYNQLLGGISREAGTEKIKGLNEVLNLAIQKNDETAHIIASLPHRFIPLFKQILSDRNTLSFILEEFKSD
EEVIQSFCKYKTLLRNENVLETAEALFNELNSIDLTHIFISHKKLETISSALCDHWDTLRNALYERRISELTGKITKSAKEKVQ
RSLKHEDINLQEIISAAGKELSEAFKQKTSEILSHAHAALDQPLPTTLKKQEEKEILKSQLDSLLGLYHLLDWFAVDESNEVD
PEFSARLTGIKLEMEPSLSFYNKARNYATKKPYSVEKFKLNFQMPTLASGWDVNKEKNNGAILFVKNGLYYLGIMPKQK
GRYK

ALSFEPTEKTSEGFDKMYYDYFPDAAKMIPKCSTQLKAVTAHFQTHTTPILLSNNFIEPLEITKEIYDLNNPEKEPKKFQTA
YAKKTGDQKGYREALCKWIDFTRDFLSKYTKTTSIDLSSLRPSSQYKDLGEYYAELNPLLYHISFQRIAEKEIMDAVETGKLY
LFQIYNKDFAKGHHGKPNLHTLYWTGLFSPENLAKTSIKLNGQAELFYRPKSRMKRMAHRLGEKMLNKKLKDQKTPIP
DTLYQELYDYVNHRLSHDLSDEARALLPNVITKEVSHEIIKDRRFTSDKFFFHVPITLNYQAANSPSKFNQRVNAYLKEHP
ETPIIGIDRGERNLIYITVIDSTGKILEQRSLNTIQQFDYQKKLDNREKERVAARQAWSVVGTIKDLKQGYLSQVIHEIVDL
MIHYQAVVVLENLNFGFKSKRTGIAEKAVYQQFEKMLIDKLNCLVLKDYPAEKVGGVLNPYQLTDQFTSFAKMGTQSG
FLFYVPAPYTSKIDPLTGFVDPFVWKTIKNHESRKHFLEGFDFLHYDVKTGDFILHFKMNRNLSFQRGLPGFMPAWDIVF
EKNETQFDAKGTPFIAGKRIVPVIENHRFTGRYRDLYPANELIALLEEKGIVFRDGSNILPKLLENDDSHAIDTMVALIRSVL
QMRNSNAATGEDYINSPVRDLNGVCFDSRFQNPEWPMDADANGAYHIALKGQLLLNHLKESKDLKLQNGISNQDWL
AYIQELR

NPKKKRKVGGYPYDVPDYA

Band 3 (C)

MKIHHHHHHEEGKLVIWINGDKGYNGLAEVGKKFEKDTGIKVTVEHPDKLEEKFPQVAATGDGPDIIFWAHDRFGGY
AQSGLLAEITPDKAFQDKLYPFTWDAVRYNGKLIAYPIAVEALSLIYNKDLLPNPPKTWEEIPALDKELKAKGKSALMFNL
QEPYFTWPLIAADGGYAFKYENGKYDIKDVGVDNAGAKAGLTFLVDLIKNKHMNADTDYSIAEAAFNKGETAMTINGP
WAWSNIDTSKVNYGVTVLPTFKGQPSKPFVGVLSAGINAASPNKELAKEFLENYLLTDEGLEAVNKDKPLGAVALKSYE
EELAKDPRIAATMENAQKGEIMPNIPQMSAFWYAVRTAVINAASGRQTVDEALKDAQTNSITSLYKKAGFMGAPPKK
KRK

VGG

MTQFEGFTNLYQVSKTLRFELIPQGKTLKHIQEQGFIEEDKARNDHYKELKPIIDRIYKTYADQCLQLVQLDWENLSAAID
SYRKEKTEETRNALIEEQATYRNAIHDYFIGRTDNLTDAINKRHAEIYKGLFKAELFNGKVLKQLGTVTTTEHENALLRSFD
KFTTYFSGFYENRKNVFSAEDISTAIPHRIVQDNFPKFKENCHIFTRLITAVPSLREHFENVKKAIGIFVSTSIEEVFSFPFYN
QLLTQTQIDLYNQLLGGISREAGTEKIKGLNEVLNLAIQKNDETAHIIASLPHRFIPLFKQILSDRNTLSFILEEFKSDEEVIQS
FCKYKTLLRNENVLETAEALFNELNSIDLTHIFISHKKLETISSALCDHWDTLRNALYERRISELTGKITKSAKEKVQRSLKHE
DINLQEIISAAGKELSEAFKQKTSEILSHAHAALDQPLPTTLKKQEEKEILKSQLDSLLGLYHLLDWFAVDESNEVDPEFSAR
LTGIKLEMEPSLSFYNKARNYATKKPYSVEKFKLNFQMPTLASGWDVNKEKNNGAILFVKNGLYYLGIMPK

QKGRYKALSFEPTEKTSEGFDKMYYDYFPDAAKMIPKCSTQLKAVTAHFQTHTTPILLSNNFIEPLEITKEIYDLNNPEKEP
KKFQTAYAKKTGDQKGYREALCKWIDFTRDFLSKYTKTTSIDLSSLRPSSQYKDLGEYYAELNPLLYHISFQRIAEKEIMDA
VETGKLYLFQIYNKDFAKGHHGKPNLHTLYWTGLFSPENLAKTSIKLNGQAELFYRPKSRMKRMAHRLGEKMLNKKLK
DQKTPIPDTLYQELYDYVNHRLSHDLSDEARALLPNVITKEVSHEIIKDRRFTSDKFFFHVPITLNYQAANSPSKFNQRVN
AYLKEHPETPIIGIDRGERNLIYITVIDSTGKILEQRSLNTIQQFDYQKKLDNREKERVAARQAWSVVGTIKDLKQGYLSQVI
HEIVDLMIHYQAVVVLENLNFGFKSKRTGIAEKAVYQQFEKMLIDKLNCLVLKDYPAEKVGGVLNPYQLTDQFTSFAKM
GTQSGFLFYVPAPYTSKIDPLTGFVDPFVWKTIKNHESRKHFLEGFDFLHYDVKTGDFILHFKMNRNLSFQRGLPGFMP
AWDIVFEKNETQFDAKGTPFIAGKRIVPVIENHRFTGRYRDLYPANELIALLEEKGIVFRDGSNILPKLLENDDSHAIDTM
VALIRSVLQMRNSNAATGEDYINSPVRDLNGVCFDSRFQNPEWPMDADANGAYHIALKGQLLLNHLKESKDLKLQNG
ISNQDWLAYIQELRNPKKKRKVGGYPYDVPDYA

A

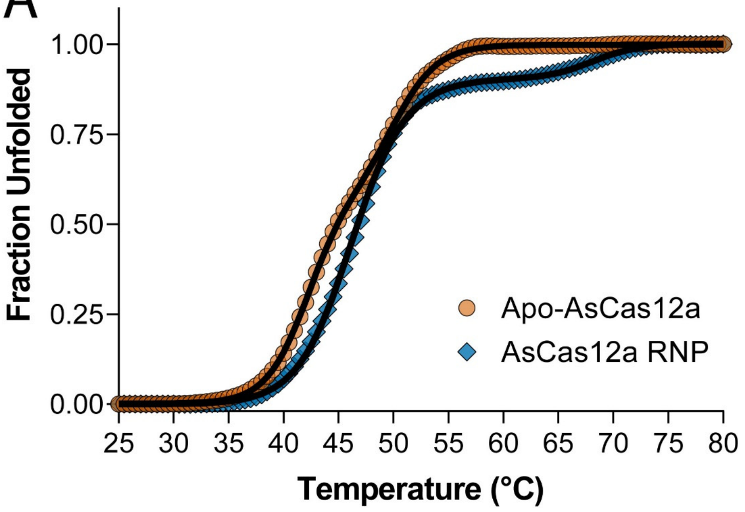

Fit values for the sequential three state (folded – intermediate – unfolded) model

	$T_{m,1}$ (°C)	$\Delta H_{vH,1}$ (kcal mol^{-1})	$T_{m,2}$ (°C)	$\Delta H_{vH,2}$ (kcal mol^{-1})
**Cas12a**	42.2 ± 0.2	-103.3 ± 2.2	50.2 ± 0.3	-105.7 ± 9.8
**Cas12a-RNP**	46.2 ± 0.2	-82.1 ± 1.4	68.2 ± 1.1	-113.9 ± 4.3

95% confidence intervals (2 x STD) reported

B

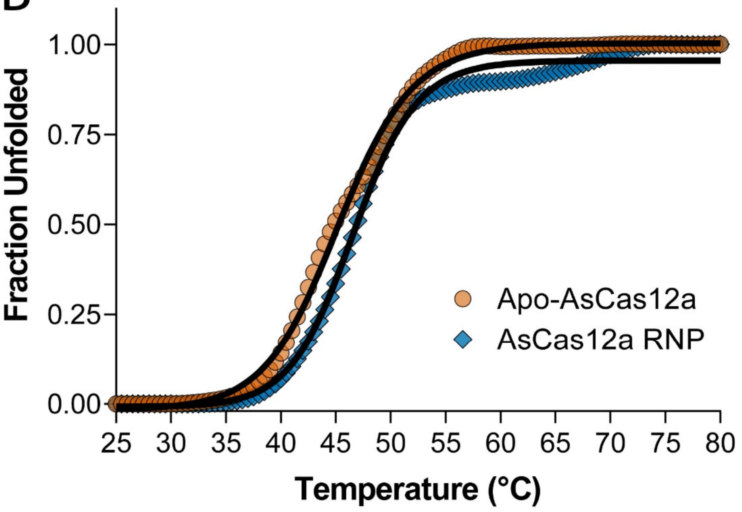

Fit values for the two state (folded -- unfolded) model

	$T_{m,1}$ (°C)	$\Delta H_{vH,1}$ (kcal mol^{-1})
**Cas12a**	42.2 ± 0.2	-103.3 ± 2.2
**Cas12a-RNP**	46.2 ± 0.2	-82.1 ± 1.4

95% confidence intervals (2 x STD) reported

Figure S23. Model fitting of DSF thermal melt data for apo-AsCas12a and AsCas12a RNP. (**A**) Three-state sequential unfolding model and (**B**) two-state unfolding model.
